# Drought-Induced Accumulation of Root Exudates Supports Post-drought Recovery of Microbes in Mountain Grassland

**DOI:** 10.3389/fpls.2018.01593

**Published:** 2018-11-07

**Authors:** Stefan Karlowsky, Angela Augusti, Johannes Ingrisch, Mohammad Kamal Uddin Akanda, Michael Bahn, Gerd Gleixner

**Affiliations:** ^1^Max Planck Institute for Biogeochemistry, Jena, Germany; ^2^Research Institute on Terrestrial Ecosystems, Consiglio Nazionale delle Ricerche, Rome, Italy; ^3^Institute of Ecology, University of Innsbruck, Innsbruck, Austria

**Keywords:** plant-soil (belowground) interactions, stress tolerance, mountain grassland, ^13^C pulse labeling, carbohydrates, NLFA, PLFA, chloroform fumigation extraction

## Abstract

Droughts strongly affect carbon and nitrogen cycling in grasslands, with consequences for ecosystem productivity. Therefore, we investigated how experimental grassland communities interact with groups of soil microorganisms. In particular, we explored the mechanisms of the drought-induced decoupling of plant photosynthesis and microbial carbon cycling and its recovery after rewetting. Our aim was to better understand how root exudation during drought is linked to pulses of soil microbial activity and changes in plant nitrogen uptake after rewetting. We set up a mesocosm experiment on a meadow site and used shelters to simulate drought. We performed two ^13^C-CO_2_ pulse labelings, the first at peak drought and the second in the recovery phase, and traced the flow of assimilates into the carbohydrates of plants and the water extractable organic carbon and microorganisms from the soil. Total microbial tracer uptake in the main metabolism was estimated by chloroform fumigation extraction, whereas the lipid biomarkers were used to assess differences between the microbial groups. Drought led to a reduction of aboveground versus belowground plant growth and to an increase of ^13^C tracer contents in the carbohydrates, particularly in the roots. Newly assimilated ^13^C tracer unexpectedly accumulated in the water-extractable soil organic carbon, indicating that root exudation continued during the drought. In contrast, drought strongly reduced the amount of ^13^C tracer assimilated into the soil microorganisms. This reduction was more severe in the growth-related lipid biomarkers than in the metabolic compounds, suggesting a slowdown of microbial processes at peak drought. Shortly after rewetting, the tracer accumulation in the belowground plant carbohydrates and in the water-extractable soil organic carbon disappeared. Interestingly, this disappearance was paralleled by a quick recovery of the carbon uptake into metabolic and growth-related compounds from the rhizospheric microorganisms, which was probably related to the higher nitrogen supply to the plant shoots. We conclude that the decoupling of plant photosynthesis and soil microbial carbon cycling during drought is due to reduced carbon uptake and metabolic turnover of rhizospheric soil microorganisms. Moreover, our study suggests that the maintenance of root exudation during drought is connected to a fast reinitiation of soil microbial activity after rewetting, supporting plant recovery through increased nitrogen availability.

## Introduction

Climate change threatens the functioning of terrestrial ecosystems, which will very likely suffer from more frequent extreme events induced by the ongoing global warming (IPCC, 2012). A large part of the terrestrial biosphere consists of grassland ecosystems that cover approximately 40% of the vegetated land surface and strongly contribute to soil carbon storage (White et al., 2000). The functioning of grasslands and their role in the global carbon cycle are particularly placed at risk by periods of severe drought (Reichstein et al., 2013; Frank et al., 2015). Grasslands in some areas may experience more severe drought effects, such as, for example, in the European Alps, which are affected by faster temperature increases compared to the global average (Beniston, 2005; Auer et al., 2007).

Extreme droughts typically lead to reduced carbon assimilation in plants (Huang and Fu, 2000; Naudts et al., 2011; Roy et al., 2016; Ingrisch et al., 2018) and reduced carbon transfer to the roots and the rhizosphere (Fuchslueger et al., 2014a, 2016; Hasibeder et al., 2015; Karlowsky et al., 2018), resulting in a lower soil CO_2_ efflux (Ruehr et al., 2009; Barthel et al., 2011; Burri et al., 2014). Consequently, the reduced belowground carbon allocation (BCA) weakens plant–microbial interactions (Brüggemann et al., 2011). Because soil microorganisms strongly depend on plant-derived carbon inputs (Wardle et al., 2004; Bardgett et al., 2005), important soil functions, such as the microbial mineralization of nitrogen and phosphorous, are limited during drought (Stark and Firestone, 1995; Borken and Matzner, 2009; Delgado-Baquerizo et al., 2013; Fuchslueger et al., 2014b; Canarini and Dijkstra, 2015; Dijkstra et al., 2015). In addition, symbiotic interactions with arbuscular mycorrhizal (AM) fungi, which strongly increase the drought resistance of plants (Allen, 2007), are affected by severe drought (Karlowsky et al., 2018). So far, whether the weakening of the link between plants and soil microorganisms during drought (i.e., the reduced soil microbial usage of recently assimilated plant-derived carbon) is due to (1) the altered carbon allocation of plants leading to reduced root exudation, (2) the limited substrate mobility in the rhizosphere, or (3) a slowdown of soil microbial metabolism is unknown. Possibly, these three mechanisms appear at the same time and interact with each other.

Drought has been shown to induce a shift of carbon allocation from the aboveground to the belowground plant organs (Palta and Gregory, 1997; Huang and Fu, 2000; Burri et al., 2014) and to increase the amounts of soluble sugars in the roots (Hasibeder et al., 2015; Karlowsky et al., 2018). The latter two studies also showed that drought-induced reductions of storage sugar concentrations are more pronounced in shoots than roots. The increase of soluble root sugars has been attributed either to osmotic regulation to support the survival of root biomass (Sicher et al., 2012; Hasibeder et al., 2015) while maintaining the carbon demand for respiration (Barthel et al., 2011) or to increased fine root growth to enhance plant access to deeper soil water resources (Huang and Fu, 2000; Burri et al., 2014). Until now, whether these drought-reduced changes in plant carbon allocation to stored reserve sugars versus soluble root sugars that are linked to exudation are affecting the carbon released into the rhizosphere has been unknown. In a recent meta-analysis of the scarce existing literature, Preece and Peñuelas (2016) found that drought can have variable effects on the rhizospheric carbon release. Strikingly, the authors of this study reported a trend toward increased root exudation per gram of plant biomass (including either root and shoot biomass or shoot biomass only) under moderate drought. However, the root biomass response to drought strongly varies among the different studies (Kreyling et al., 2008 and references therein), potentially affecting the total amount of carbon released to the rhizosphere. For example, Fuchslueger et al. (2014a) found that a slightly increased root to shoot ratio during drought was mirrored by higher amounts of plant-derived carbon in the extractable organic carbon (EOC) of soil.

The drying of soil itself has major impacts on the exudate transfer from the release site to rhizospheric microorganisms, which might increase the competition for substrates between functionally different microbial groups. In contrast to AM fungi, which are directly connected to the root carbohydrate pool, saprotrophic fungi (SF) and bacteria depend on the diffusion of substrates for their nutrition (Manzoni et al., 2012). As the lower water content during drought conditions limits the diffusion of substrates (Skopp et al., 1990), the uptake of nutrients by SF and bacteria is limited. Moreover, experimental results suggest that the microbial activity in the soil depends on the environmental conditions that affect diffusion pathways between substrate sources and microorganisms (Nunan et al., 2017). Consequently, if root exudation is increased along with root growth during drought, plant-derived solutes likely will accumulate in the rhizosphere due to reduced microbial carbon mineralization. Indeed, increased amounts of dissolved organic carbon immediately after the rewetting of dried soils (Canarini et al., 2017) suggest the existence of such accumulations. These additional carbon sources could further contribute to the pulse of soil respiration, which appears after rewetting and is associated with higher soil microbial activity and nitrogen mineralization (Birch, 1958). The so-called ‘Birch effect’ is present in planted and unplanted soils (Canarini et al., 2017) and has been suggested to primarily originate from osmolytes, which accumulate in microbial cells during drought conditions (Fierer and Schimel, 2003). As a stress response to desiccation, the synthesis of microbial osmolytes is increased at the expense of membranes for cell growth (Schimel et al., 2007). To prevent the bursting of cells due to excessive water uptake, accumulated osmolytes need to be rapidly metabolized after rewetting (Warren, 2014). The metabolically active microorganisms are probably also able to use excess plant-derived carbon, which could support plant recovery by further increasing the nitrogen mineralization rate in the soil.

Plant carbon allocation is best analyzed by pulse-labeling of the plant canopy with ^13^C-enriched CO_2_ and tracing of the assimilated ^13^C by compound specific carbon isotope (^13^C/^12^C) ratios of plant non-structural carbohydrates (NSCs) (Bahn et al., 2013; Karlowsky et al., 2018). Similarly, root exudation and the subsequent microbial carbon uptake can be determined by combining the K_2_SO_4_ extraction and chloroform fumigation method (Vance et al., 1987) with ^13^C analysis (Malik et al., 2013). This allows the flow of plant-derived carbon in EOC and microbial biomass carbon (MBC) from soil to be traced. The water-soluble EOC is mainly a proxy for the exuded plant carbon (Supplementary Figure S1), with minor contributions of AM fungi exudation (Drigo et al., 2010; Balasooriya et al., 2012; Kaiser et al., 2015), which is also directly linked to the plant-derived carbon (Supplementary Figure S1). To determine the uptake of plant-derived carbon by the different soil microbial groups, compound-specific ^13^C isotope analysis on phospholipid fatty acid (PLFA) markers from soil can be used (Kramer and Gleixner, 2006). A comparison of the ^13^C incorporation into MBC and into PLFA markers allows distinctions to be made between the growth and maintenance of soil microorganisms (Malik et al., 2015).

To study the rhizospheric processes, we used a common garden experiment on a mountain meadow using species representing the local meadow community. Our main objective was to assess the effects of drought and rewetting on the response of plant–microbial carbon transfer as a fundamental part of ecosystem functioning (Wardle et al., 2004; Bardgett et al., 2005; Schimel et al., 2007; Brüggemann et al., 2011). We performed two ^13^C pulse chase campaigns, a first at peak drought and second shortly after rewetting, and studied the response of carbon assimilation, allocation and transfer to soil microbial markers.

Specifically, we hypothesized that the weakening of the link between plant and soil processes during drought is mainly due to decreased transfer of microbial carbon substrates in the rhizosphere and osmotic effects and is not due to decreased carbon release from roots increasing the competition for carbon between microorganisms. Furthermore, we expected that drought would lead to an accumulation of root sugars and easily degradable EOC in soil, which are available for priming plant and soil microbial activity after rewetting.

## Materials and Methods

### Experimental Site

The study site is near Neustift in the Stubai Valley in the Austrian Central Alps (1,820–1,850 m a.s.l.; 47°7′45^′′^N, 11°18′20^′′^E) and is described in Bahn et al. (2009). Briefly, the average annual temperature is 3°C, the annual precipitation is 1,097 mm, and the soil is a dystric cambisol type. The site is a hay meadow that is cut once per year at peak biomass in early August, is lightly manured every 2–3 years, and has a Trisetum flavescentis vegetation type consisting of perennial grasses and forbs (Schmitt et al., 2010). The meadow soil has a loamy sand texture and a bulk density of 0.7 g cm^-3^ (Meyer et al., 2012a). The total soil carbon content in the uppermost 10 cm is 51 g kg^-1^ (Meyer et al., 2012b).

### Establishment of Mesocosms

In 2013, a replicated mesocosm experiment with six blocks and eight mesocosms per block was established on the experimental site. For each mesocosm, two dark plastic pots, 45 cm in diameter and 35 cm in height, one inside the other, were used. The external pot was used as water reservoir and the internal one was used to hold the soil and the plants. Each pot was filled with sieved (<5 mm) subsoil (below 10 cm) from the study site and embedded in the soil on the experimental site. To prevent a possible impact from runoff water on the experiment, the upper edge of the mesocosms were raised by 2 cm relative to the soil surface. A representative selection of plant species from the site was chosen, which consisted of grass, forb and legume species. The individual plants (shoots and roots) were excavated at the experimental site in early July 2013 and were pre-incubated for 6–7 weeks in a greenhouse, in the botanical garden of Innsbruck, Austria. Every mesocosm was planted in late August 2013 with three grasses (*Deschampsia cespitosa, Festuca rubra*, and *Dactylis glomerata*), two forbs (*Leontodon hispidus* and *Geranium sylvaticum*) and one legume (*Trifolium repens*). At the time of planting, the plant shoots had a height of 5–15 cm. All mesocosms were planted with 36 individuals and with varying relative abundances of the different grass and forb species (Supplementary Table S1). The amount of the legume remained constant to exclude a possible nitrogen fertilization effect. The position of individual plants was randomized on a fixed pattern of locations for each mesocosm. All mesocosms were randomized in the block design. In 2014, the plant community was established on the site, and the biomass was harvested according to the common practice on August 22nd, 2014.

### Drought Treatment and Pulse Labeling

The experiment began on the 5th of June 2015 by simulating early summer drought (Supplementary Figure S2A), similar to the method described by Ingrisch et al. (2018) and Karlowsky et al. (2018) for a common garden experiment with intact vegetation-soil monoliths. In brief, six rain-out shelters (Supplementary Figure S2B), with base areas of 3 m × 3.5 m and 2.5 height, covered by light- and UV-B permeable plastic foil (Lumisol clear AF, Folitec, Westerburg, Germany, light transmittance c. 90%), were installed above the mesocosms. Air ventilation was maintained with an opening the bottom (<0.5 m above ground) and at the top of the sides of the rain-out shelters, thereby preventing the entrance of rain water. On a subset of four to five mesocosms per shelter, soil water content (SWC) and temperature were monitored continuously in the main rooting horizon [5TM sensors (*n* = 17) for combined SWC and temperature measurement and EC-5 sensors (*n* = 11) for SWC measurement, connected to Em50 loggers; Decagon Devices, Pullman, WA, United States]. In addition, the SWC was measured manually for each mesocosm with a PR2 Soil Moisture Profile Probe (Delta-T Devices Ltd., Cambridge, United Kingdom) at depths of 5 cm and 15 cm between the 12th of June and the 10th of August (13 times during drought and four times during recovery).

During rain exclusion, the mesocosms of the control treatments were watered manually to SWCs greater than 19% to avoid water limitation. No water was given to drought-treated mesocosms, yielding SWCs of approximately 6 and 10% at depths of 5 and 15 cm, respectively, at peak drought (Supplementary Figure S3). Soil moisture at field capacity was estimated on the 1st of June 2018 on the same mesocosms as 38.6% (*SD* = 6.7%, *n* = 27) using data (from 5TM and EC-5 sensors) collected when the soil moisture had stabilized a few days after rain. Four weeks after the drought treatment started, the first ^13^C pulse labeling (peak drought labeling) started on the 4th of July on a subset of 12 mesocosms (six control and six drought treatments). Drought simulation was stopped on the 14th of July 2015, by removing the rain-out shelters and adding water representing 25 mm of precipitation to all mesocosms (control and drought treatments). Because of a natural dry period, from the 15th to the 22nd of July, another 16 and 36 mm of precipitation equivalents were added in total to the control and drought treatments, respectively. On a subset of another 12 mesocosms, after a recovery phase of 10 days, the second ^13^C pulse labeling (recovery labeling) began on the 24th of July.

Both labeling campaigns were done on three consecutive days (peak drought from the 4th until the 6th of July; recovery from the 24th until the 26th of July) with high radiation. For each labeling campaign, one control and one drought mesocosm were used in each of the six rain-out shelters (Supplementary Figure S2C). The ^13^C pulse labeling was done on 2–6 mesocosms per day. The labeling was always done in parallel on one drought mesocosm and one control mesocosm, with the starting time shifted by 15 min (randomly started with either control or drought mesocosm). Because the plant growth strongly varied between mesocosms from the same planting scheme, we aimed to visually choose pairs of mesocosms that were as similar as possible. Pulse labeling was performed similarly, as described by Bahn et al. (2009, 2013) and Hasibeder et al. (2015). Briefly, a cylindrical and transparent Plexiglas chamber with 45-cm diameter and 50-cm height was placed on the top of the mesocosms with a rubber gasket between the chamber and the mesocosm (Supplementary Figure S2D). Elastic bands were used to fix the chamber on external anchor points in order to ensure gas tightness. Air circulation and temperature control were handled by fans and tubes connected to a pump circulating water cooled with ice packs. During the pulse labeling, we monitored the interior air temperature (shaded sensor), CO_2_ concentration (Licor 840A, Lincoln, NE, United States) and ^13^C isotope ratio of CO_2_ (Picarro G2201i Analyzer, Picarro Inc., Santa Clara, CA, United States). Solar radiation was measured outside the chamber using a PAR quantum sensor (PQS 1; Kipp & Zonen, Delft, Netherlands). Pulse labeling was done under comparable light conditions on mostly clear days between 10:00 and 15:00 CET. Highly enriched ^13^CO_2_ (>99 atom% ^13^C; Sigma-Aldrich, Taufkirchen, Germany) was added pulse-wise to achieve 30–80 atom% ^13^C in chamber CO_2_ over the complete labeling time of 75 min (peak drought labeling) and 30 min (recovery labeling). The CO_2_ concentrations were, on average, 568 ± 99 ppm and 671 ± 98 ppm during the peak drought and the recovery labeling campaigns, with some variation caused by the pulse-wise addition of ^13^CO_2_ (Supplementary Table S2). Potential effects of species-specific differences in isotopic fractionation under slightly elevated CO_2_ or drought on recovered amounts of ^13^C can be excluded due to the significant enrichment of ^13^C from naturally 1.1 to 30–80 atom% during the labeling campaigns.

### Sampling

For each mesocosm, plant and soil samples were collected in a time series after the pulse labeling. The time series included samplings at 15 min, 24, 72, and 120 h after the labeling chamber was removed. Because a minimum distance of ∼5 cm had to be kept to the mesocosm edge, to a soil moisture measurement site and to a centrally located soil respiration measurement chamber, the available area for plant and soil sampling was very limited. The first sampling location was randomly chosen in the available area and further samplings were performed either clockwise or counterclockwise in a distance of ∼5 cm. At each sampling, the shoot material, i.e., the leaves and stems, was cut 1 cm above the soil in two 5 cm × 5 cm squares, which included a random selection of plant species from opposite positions in the mesocosm. The shoot material from both squares was pooled together and separated into biomass and necromass. The biomass was immediately treated by microwave to interrupt any metabolic activity (Popp et al., 1996), stored on ice packs for transport and dried at 60°C for 72 h for later analysis of the sugar content and stable carbon isotope composition. For soil samples, soil cores were collected in or next to plant sampling squares on bare soil spots close to plant cover. Sampling was done using a stainless-steel auger with 1.9 cm inner diameter (Eijkelkamp, Giesbeek, Netherlands). At each sampling, four soil cores (two per shoot sampling square) were taken from a depth of 0–7 cm and pooled in a mixed sample. Mixed soil samples were carefully sieved through a 2-mm mesh, and the roots were removed. Soil for EOC and MBC analysis was transported on ice packs, stored at 4°C and extracted/fumigated by no later than 4 days after sampling. Soil for neutral/phospho-lipid fatty acid (NLFA/PLFA) analysis was directly frozen with dry ice and stored at -18°C until further preparation. Subsamples of frozen soil were used prior to the NLFA/PLFA analysis to determine the soil water content gravimetrically, by weighing the soil before and after drying for 48 h at 105°C. Roots were washed from the remaining soil, and the dead as well as coarse roots (diameter > 2 mm) were removed. The total amount of washed fine root samples was divided into two subsamples. One subsample was treated like shoot samples (microwaved), and the other one (not microwaved) was kept moist with wet paper towels and used as quickly as possible for root respiration measurements in the field.

Microwaved shoot and root samples were completely dried in an oven at 60°C for 72 h, starting on the day of harvest. After its dry weight had been determined, the plant material was carefully ground to a fine powder using a ball mill (MM200, Retsch GmbH, Haan, Germany). This material was then used to analyze the bulk ^13^C content, the compound-specific ^13^C isotope composition and the bulk nitrogen concentration. The aboveground biomass of the mesocosms was harvested completely at the end of each labeling/sampling campaign to determine the community shoot biomass. Community root biomass was directly estimated from the dry mass of all root samples for each individual mesocosm. To obtain samples with natural ^13^C abundance, on the 14th of July, one soil core was taken from each of four unlabeled control mesocosms, and these cores were pooled together. The same was done for the unlabeled drought mesocosms. Similarly, shoot material was collected from all six species of each mesocosm and pooled together for the four control and four drought mesocosms.

### Isotopic Composition of Plant Samples and Carbohydrate Analysis

Ground bulk plant material was used to determine ^13^C contents (δ^13^C vs. VPDB) and nitrogen concentrations of shoots and fine roots by elemental analysis (EA) – isotope ratio mass spectrometry (IRMS) (EA - Model NA 1500, Carlo Erba, Milan, Italy; coupled to an IRMS IsoPrime100, Isoprime Ltd., Cheadle, United Kingdom). NSC analysis was done as described by Karlowsky et al. (2018). Briefly, 30 mg of plant powder was weighed, and water-soluble sugars (fructan, sucrose, glucose, and fructose) were extracted using the method of Wild et al. (2010), as modified by Mellado-Vázquez et al. (2016). Analysis was done by high-performance liquid chromatography (HPLC) – IRMS (Dionex UltiMate 3000 UHPLC coupled via a LC-IsoLink system to a Delta V Advantage IRMS, Thermo Fisher Scientific, Bremen, Germany) in a NUCLEOGEL SUGAR 810 Ca^2+^ column (Macherey & Nagel, Düren, Germany) at 80°C, with 0.5 ml/min of bi-distilled water as eluent (Hettmann et al., 2007). In accordance with previous findings from the same study site (Karlowsky et al., 2018), fructan was assigned to one large peak at the beginning of chromatograms, which likely represented fructans with a high degree of polymerization (Benot et al., 2013). For starch analysis, the remaining pellets from the sugar extraction were washed again with a methanol:chloroform:water mixture (12:3:5, by volume) to remove remaining sugars and then digested with heat stable α-amylase (Göttlicher et al., 2006; Richter et al., 2009). The resulting gluco-oligomers were measured by EA-IRMS (EA 1100, CE Elantech, Milan, Italy; coupled to a Delta + IRMS, Finnigan MAT, Bremen, Germany).

### Root Respiration Measurements

A subsample (0.2–1.2 mg) of root material, washed from soil and kept moist, was used for root respiration measurement in the field. Fresh roots were placed in a 100-ml Erlenmeyer flask, sealed by a rubber stopper and incubated at 15 ± 1°C in a water bath. The initial CO_2_ concentration in the flask was, on average, 491 ± 12 ppm. Root incubation was performed according to Hasibeder et al. (2015), except for the time collection. Specifically, five gas samples were collected: one immediately after closing the flask and the other four after 7, 20, 40, and 60 min, respectively. Gas sampling was performed with a syringe; each time, 15 ml of gas was collected and transferred completely into pre-evacuated 12 ml vials with a rubber septum, to prevent ambient air from entering the vial. After each sampling, 15 ml CO_2_-free air was injected into the Erlenmeyer flasks to replace the gas collected. The CO_2_ concentration and the ^13^C isotope composition were analyzed by IRMS coupled with a Multiflow system (IsoPrime100, Isoprime Ltd., Cheadle, United Kingdom). All gas samples were analyzed as soon as possible after sampling and were stored in the laboratory for a maximum of 4 weeks. Root respiration rate and the ^13^C/^12^C ratio of the CO_2_ respired were calculated according to Hasibeder et al. (2015).

### Analysis of Soil-Extractable Organic Carbon and Microbial Biomass Carbon

For the determination of the soil EOC and MBC, the method of Vance et al. (1987) with the modifications of Malik et al. (2013), was used. Soil EOC was extracted from a subsample of approximately 5 g of fresh soil with 25 ml of 0.5 M K_2_SO_4_ solution (distilled water) in a horizontal shaker with 150 rpm for 30 min. The extract was centrifuged at 12,000 × *g* for 5 min and coarse particles were removed using pre-washed (0.5 M K_2_SO_4_ solution) filter papers (Whatman Grade 1, *d* = 150 mm, 11 μm pore size, GE Healthcare UK Ltd., Buckinghamshire, United Kingdom). The filtrate was frozen and stored at -18°C until further processing for analysis. Total organic carbon (TOC) was extracted and processed in the same way as the EOC, after another subsample of approximately 5 g fresh soil had been fumigated for ≥24 h with chloroform. If necessary, drought-treated soils were rewetted to control levels with distilled water prior to the fumigation to avoid differences in the extraction efficiency (Sparling et al., 1990). For the analysis, ∼1 ml each of the EOC and TOC extracts was filtered with pre-washed (∼0.5 ml of extract) 0.45 μm cellulose membrane filters (MULTOCLEAR 0.45 μm RC 13 mm, CS-Chromatographie Service GmbH, Langerwehe, Germany). To de-gas the samples of inorganic C, filtered extracts were acidified with phosphoric acid to approximately pH 2 and gas-flushed with N_2_ for 15 min. The degassed samples were then analyzed as bulk fraction (no column) on an HPLC-IRMS system (see carbohydrate analysis). Each sample was measured in triplicate. Quality was controlled by repeated measurements of citric acid standards (δ^13^C = -18.58 ‰ vs. VPDB, Fluka Chemie AG, Buchs, Switzerland; *SD* = 0.14‰, *n* = 72). Quantification was performed using a concentration row of the citric acid standard to calibrate the HPLC-IRMS based on CO_2_ peak areas. The results for the EOC and TOC were normalized to the used soil dry mass for each fraction, and the concentration of MBC was calculated from the EOC and TOC by the formula: [MBC] = ([TOC] - [EOC])/*k*_MBC_. For *k*_MBC_, a value of 0.45 was used, which is the typical extraction efficiency of MBC after chloroform fumigation (Vance et al., 1987). The ^13^C/^12^C ratio (i.e., δ^13^C or atom% ^13^C) of MBC was calculated according to the isotopic mass balance: ^13^C/^12^C_MBC_ = (^13^C/^12^C_TOC_^∗^ [TOC] -^13^C/^12^C_EOC_^∗^ [EOC])/([TOC]-[EOC]).

### Analysis of Neutral and Phospholipid Fatty Acids

Neutral and phospholipid fatty acid analysis was done according to the method of Bligh and Dyer (1959), as modified by Karlowsky et al. (2018). Briefly, approximately 5 g of frozen bulk soil was extracted with a mixture of methanol, chloroform and 0.05 M K_2_HPO_4_ buffer (2:1:0.8, by volume; pH 7.4) using pressurized solvent extraction (SpeedExtractor E-916, Büchi Labortechnik AG, Flawil, Switzerland). A recovery standard (1,2-Dinonadecanoyl-sn-Glycero-3-Phosphatidylcholine; Larodan Fine Chemicals AB, Malmö, Sweden) was added (recovery rate: 62 ± 11%, SD, *n* = 60) to each sample, and the extraction was carried out at 70°C and 120 bar for 3 min × 10 min. Neutral and phospholipid fractions were separated using silica-filled solid-phase extraction (SPE) columns (CHROMABOND SiOH, 2 g, 15 ml, MACHEREY-NAGEL GmbH & Co. KG, Düren, Germany). Both fractions were hydrolyzed and methylated with methanolic KOH, and the resulting fatty acid methyl esters (FAMEs) were further purified for analysis by using aminopropyl-modified SPE columns (CHROMABOND NH2, 0.5 g, 3 ml, MACHEREY-NAGEL GmbH & Co. KG, Düren, Germany). The FAME C13:0 (Sigma-Aldrich Chemie GmbH, Munich, Germany) was added as the internal standard to all samples, and quantification was done by gas chromatography–flame ionization detection (GC-FID) on a GC-FID 7890B system with a programmable temperature vaporization (PTV) injector (Agilent Technologies, Palo Alto, CA, United States) using a DB-1MS UI column (30 m × 0.25 mm internal diameter × 0.25 μm film thickness, Agilent Technologies, Palo Alto, CA, United States) and helium as the carrier gas (1.8 ml/min). The temperature program started at 45°C for 1 min, then increased in a first ramp of 60°C/min to 140°C (held for 0.5 min), followed by a second ramp of 2°C/min until 242°C, and finally, by a third ramp to 320°C (held for 3 min). Directly after injection, the PTV was heated up from 55 to 280°C at a rate of 500°C/min. Compound specific ^13^C isotope analysis of NLFAs and PLFAs was conducted by GC-IRMS (GC 7890A with PTV injector, Agilent Technologies, Palo Alto, CA, United States; coupled via a Conflo IV/GC IsoLink to a Delta V Plus IRMS, Thermo Fisher Scientific, Bremen, Germany) using a DB-1MS UI column (60 m × 0.25 mm internal diameter × 0.25 μm film thickness, Agilent Technologies, Palo Alto, CA, United States) and helium as the carrier gas (1.8 ml/min). Directly after injection, the PTV was heated from 55 to 280°C at a rate of 500°C/min. The GC temperature program started with 45°C for 1 min, then increased in a first ramp of 60°C/min to 140°C (held for 0.5 min), followed by a second ramp of 4°C/min until 283°C (held for 4.9 min) and a third ramp until 320°C (held for 3 min). Concentrations and ^13^C isotope content of identified FAMEs were corrected for the methyl group introduced during derivatization. We used the sum of the PLFAs i14:0, i15:0, a15:0, i16:0, a17:0, i17:0, and br18:0 for Gram-positive bacteria (Zelles, 1997, 1999); 10-Me16:0 and 10-Me18:0 for actinobacteria (Lechevalier et al., 1977; Zelles, 1999); and 16:1ω7 and 18:1ω7 for Gram-negative bacteria (Zelles, 1997, 1999). The PLFA 18:2ω6,9 was used as the marker for saprotrophic fungi (Frostegård and Bååth, 1996; Zelles, 1997) and the NLFA 16:1ω5 as the marker for arbuscular mycorrhizal (AM) fungi (Olsson, 1999). Although the NLFA 16:1ω5 does not correctly estimate the biomass of AM fungal populations, it has been found to be more of a proxy than the PLFA 16:1ω5 (e.g., Ngosong et al., 2012; Mellado-Vázquez et al., 2016; Paterson et al., 2016).

### Calculation of ^13^C Tracer Concentrations

To determine the relative abundance of ^13^C tracer in labeled samples, we calculated the atom% ^13^C_excess_ as follows:


atom% C13excess=atom% C13labeled−atom% C13unlabeled

with *atom% ^13^C_labeled_* being the atom% ^13^C of the labeled samples and *atom% ^13^C_unlabeled_* being the atom% ^13^C of natural abundance samples from unlabeled mesocosms (mixed samples from shoots of all six species were used as reference for the plant community). Values of atom% ^13^C_excess_ are not presented here but can be found in the Supplementary Figures S9–S12.

For all plant and soil samples, we expressed the ^13^C isotope content as incorporated ^13^C (mg ^13^C m^-2^), which refers to the total amount of ^13^C found in a certain carbon pool on an area basis, and it was calculated as:


incorporatedC13=atom%C13excess∗Cpool100%

with *C*_pool_ being the respective carbon pool (mg C m^-2^).

The roots respired ^13^C (mg ^13^C m^-2^ h^-1^), which corresponds to the amount of ^13^C released in respired CO_2_ from roots during a certain time, was calculated similarly to the incorporated ^13^C as follows:


root respiredC13=atom%C13excess∗CO2resp.rate100%

with *CO*2_resp.rate_ being the respiration rate of CO_2_ (mg CO_2_ m^-2^ h^-1^).

### Data Analyses

For root biomass and concentration data, the average values were calculated over the different sampling times after pulse labeling: 1 and 3 days after labeling for NLFAs and PLFAs and 15 min, 1 day, 3 days, and 5 days after labeling for all others. For the soil samples, a bulk soil density of 0.7 g cm^-3^ (Meyer et al., 2012a) was used for calculating area-based pool sizes. The total ^13^C uptake was calculated as the sum of the bulk shoot and bulk root incorporated ^13^C at the first sampling directly after labeling (15 min). The ^13^C tracer fluxes were analyzed for drought effects considering the different sampling times (same times as for concentration data). After removing negative ^13^C incorporation values (defined as below detection limit), the relative ^13^C allocation to the different pools was calculated for each sampling time as the ratio of ^13^C incorporation to total ^13^C uptake. Relative ^13^C allocation to shoot and root storage pools was calculated as the sum of relative ^13^C allocation to fructan and starch in the shoots and roots. For an overview of the drought effects on all pools (including NLFAs and PLFAs), the relative ^13^C allocation was averaged for 1 and 3 days samples, and the drought to control ratio was calculated. In general, at 1 and 3 days after pulse labeling, the drought effects on relative ^13^C allocation were comparable (Supplementary Figure S4) and high ^13^C tracer enrichment was found in all pools of interest, making these two times suitable to assess the strongest differences in ^13^C allocation patterns. For the calculation of drought to control ratios, only labelings with data from both treatments (i.e., control and drought mesocosms that were labeled at the same time,) were considered. First, the drought to control ratio of each labeling pair was calculated, and second, the average value was formed.

All statistical analyses were done using the R 3.3.2 software (R Core Team, 2016). Time series (in hours after pulse labeling) of the ^13^C tracer data were tested separately for each labeling campaign for the effects of drought and sampling time, as well as their interaction, using linear mixed-effects models from the ‘lme4’ package (Bates et al., 2015). In the mixed-effects model, the treatment and sampling time (as factor) were set as fixed effects, whereas the rain-out shelter and mesocosm were set as random effects. Drought effects on relative ^13^C allocation were analyzed similarly, using treatment and sampling time (as factors) as fixed effects, and labeling pair (control and drought mesocosms labeled in parallel) and mesocosm as random effects. All mixed-effects models were assessed for violations of normality, heteroscedasticity and independency. If necessary, ^13^C tracer data were log (+1) or square root (+1) transformed. For all other data (i.e., biomass, total ^13^C uptake and concentration data), the drought effects were evaluated for each labeling campaign separately using permutational ANOVA from the ‘lmPerm’ package (Wheeler and Torchiano, 2016), from which exact *P*-values (*P_aovp_*) were obtained. Permutation tests do not require assumptions about the statistical distribution and are powerful with small sample sizes (Ernst, 2004).

## Results

### Peak Drought Labeling

**FIGURE 1:**
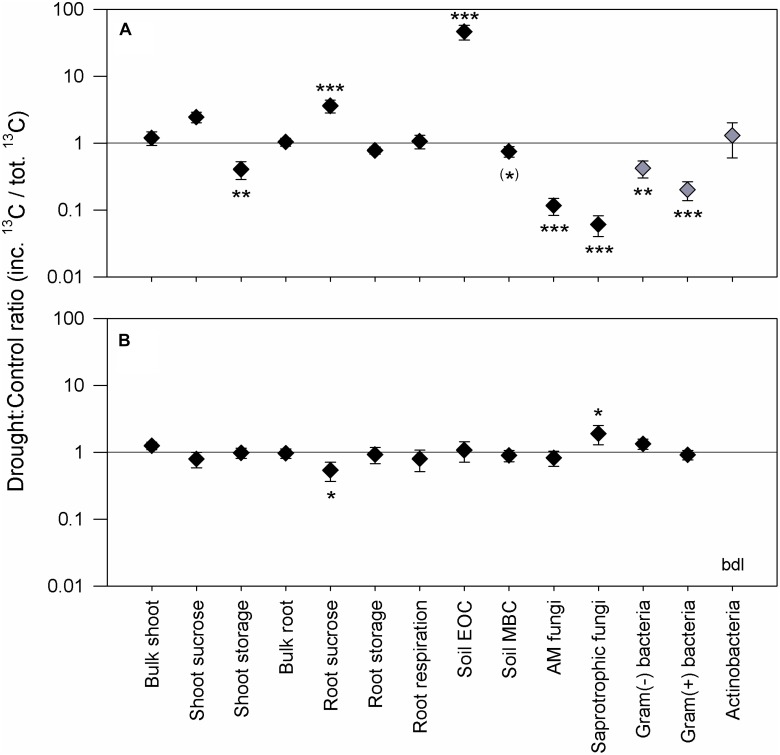
Effects of drought on C allocation patterns at the peak drought **(A)** and recovery **(B)** labeling campaigns. The drought to control ratio of the relative ^13^C allocation is shown, i.e., the amount of incorporated ^13^C (inc. ^13^C) in each pool that was recovered from the total ^13^C uptake (tot. ^13^C), averaged for the samplings at 24 and 72 h after pulse labeling. The graph only highlights the strongest effects, and additional data for individual sampling points, including 15 min and 120 h, can be found in Supplementary Figure S4. Black symbols represent the mean of *n* = 6 control/drought pairs, and gray symbols the mean of *n* = 3 control/drought pairs. Error bars were obtained by propagating the SE from the replicates of each treatment, control and drought, respectively. Asterisks indicate levels of significance for drought effects (df = 1) from the linear mixed-effects models: ^∗∗∗^*P*_χ^2^_ < 0.001, ^∗∗^*P*_χ^2^_ < 0.01, ^∗^*P*_χ^2^_ < 0.05, and ^(∗)^*P*_χ^2^_ < 0.1. The “bdl” notation stands for below detection limit.

The 4 weeks of severe drought had strong effects on the plant community and its biomass at peak drought (Table 1). Drought significantly reduced the shoot biomass but had no distinct effect on the total plant biomass, since a strong increase of fine root biomass occurred. Consequently, drought led to a significant increase in the root to shoot ratio. According to the reduction in shoot biomass, the photosynthetic rate (Supplementary Figure S5A) and total plant ^13^C uptake (Table 1) were strongly reduced by drought as well. Drought did not change the proportion of total ^13^C (relative ^13^C allocation) that was allocated belowground at 24 and 72 h from labeling (Figure 1A), although it was lower at 15 min and higher at 120 h (Supplementary Figure S4). The little effect of drought on overall BCA was also expressed by similar reductions of ^13^C tracer incorporation into shoots and roots over the 120-h sampling period (Supplementary Figure S6). However, drought more strongly affected relative ^13^C allocation to NSCs (Figure 1A) and their tracer dynamics (Supplementary Figures S6B–D,F–H). Significantly less ^13^C was allocated to shoot storage (Figure 1A), i.e., to compounds such as fructan and starch (Supplementary Figures S6C,D), whereas slightly more ^13^C was retained in shoot sucrose over time (Figure 1A and Supplementary Figure S4). This retention was reflected in the higher sucrose concentrations and lower fructan and starch concentrations in drought shoots compared to controls (Table 2). Drought increased the relative ^13^C allocation to the root sucrose pool (Figure 1A), which showed altered tracer dynamics (Supplementary Figure S6F), i.e., lower ^13^C incorporation until 24 h and higher ^13^C incorporation. Reduced ^13^C incorporation was found in fructan and starch from roots (Supplementary Figures S6G,H), although their concentrations (Table 2) were not affected by drought. Indeed, the relative ^13^C allocation to root storage was on average only little affected by drought (Figure 1A), showing a decrease at 24 h and an increase at 120 h (Supplementary Figure S4). Apparently, in root fructan, drought mainly led to slower ^13^C tracer incorporation over time (Supplementary Figure S6G). Moreover, considered the higher fine root biomass, the root fructan pool even increased during drought (Control, 6.1 ± 1.3 g_C_ m^-2^; Drought, 10.2 ± 1.5 g_C_ m^-2^; SE, *n* = 6; *P_aovp_* = 0.009). Similar to root storage, the drought reduced the amount of root-respired ^13^C but only at the first two sampling points (Supplementary Figure S7A). This reduction led to decreased relative ^13^C allocation to root respiration at 15 min and 24 h; however, it increased at 72 and 120 h (Supplementary Figure S4). This effect was not visible on average for 24 and 72 h (Supplementary Figure S1). Consequently, the overall respiration rate was not altered by drought (Table 1), despite lower respiration rates at the dry mass level (Control, 4.6 ± 0.3 nmol_CO2_ g^-1^_dm_ s^-1^; Drought, 3.3 ± 0.6 nmol_CO2_ g^-1^_dm_ s^-1^; *P_aovp_* < 0.001). Plant nitrogen concentrations were only little affected by drought and tended to be higher in shoots (Control, 1.31 ± 0.04%_N_; Drought, 1.40 ± 0.06%_N_; *P_aovp_* = 0.076) but not in roots (Control, 0.79 ± 0.05%_N_; Drought, 0.86 ± 0.06%_N_; *P_aovp_* = 0.206). However, if the differences in biomass were considered, drought led to a reduction of shoot nitrogen content and an increase of root nitrogen content per unit area (Table 1).

**Table 1:** Drought effects on biomass, ^13^C tracer uptake, root respiration and biomass N contents.

Labeling	Parameter	Unit	Control	Drought	*D* ^a^
Peak drought	Total biomass	g_dm_ m^-2^	313 ± 23	353 ± 31	n.s.
	Shoot biomass	g_dm_ m^-2^	131 ± 12	82 ± 9	^∗∗∗^
	Root biomass	g_dm_ m^-2^	182 ± 16	271 ± 25	^∗∗^
	Root:Shoot ratio	–	1.45 ± 0.21	3.44 ± 0.37	^∗∗∗^
	^13^C uptake	mg_13C_ m^-2^	366 ± 32	93 ± 6	^∗∗∗^
	Root respiration	μmol_CO2_ m^-2^ s^-1^	0.82 ± 0.03	0.88 ± 0.09	n.s.
	Shoot N	g_N_ m^-2^	1.71 ± 0.16	1.14 ± 0.13	^∗∗^
	Root N	g_N_ m^-2^	1.41 ± 0.10	2.35 ± 0.30	^∗∗∗^
	Total N	g_N_ m^-2^	3.12 ± 0.22	3.49 ± 0.38	n.s.
Recovery	Total biomass	g_dm_ m^-2^	295 ± 19	267 ± 12	n.s.
	Shoot biomass	g_dm_ m^-2^	114 ± 8	102 ± 7	n.s.
	Root biomass	g_dm_ m^-2^	181 ± 20	165 ± 8	n.s.
	Root:Shoot ratio	–	1.7 ± 0.3	1.6 ± 0.1	n.s.
	^13^C uptake	mg_13C_ m^-2^	220 ± 29	231 ± 27	n.s.
	Root respiration	μmol_CO2_ m^-2^ s^-1^	0.81 ± 0.06	0.94 ± 0.11	n.s.
	Shoot N	g_N_ m^-2^	1.34 ± 0.09	1.74 ± 0.19	^∗∗^
	Root N	g_N_ m^-2^	1.46 ± 0.19	1.59 ± 0.03	n.s.
	Total N	g_N_ m^-2^	2.80 ± 0.23	3.33 ± 0.19	^∗^


*^a^Levels of significance for drought effects: ^∗∗∗^P_aovp_ < 0.001, ^∗∗^P_aovp_ < 0.01, ^∗^P_aovp_ < 0.05, ^(∗)^P_aovp_ < 0.1; n.s., not significant. Mean values ± SE (n = 6) are shown for control and drought treatments. For root respiration and N concentrations, the data were averaged over the four sampling times for each mesocosm.*

**Table 2:** Effects of drought on the sizes of plant bulk and carbohydrate pools for the peak drought and the recovery labeling campaigns.

Labeling	Parameter	C content (mg_C_ g_dm_^-1^)		
		
		Control	Drought	*D* ^a^
Peak drought	Bulk shoot	422 ± 3	423 ± 3	n.s.
	Shoot sucrose	14 ± 0	16 ± 1	^∗∗∗^
	Shoot fructan	57 ± 2	41 ± 3	^∗∗∗^
	Shoot starch	8.1 ± 0.6	5.1 ± 1.4	^∗∗^
	Bulk root	345 ± 15	369 ± 15	^(∗)^
	Root sucrose	4.4 ± 0.4	10.8 ± 0.9	^∗∗∗^
	Root fructan	32 ± 2	38 ± 6	n.s.
	Root starch	12 ± 4	16 ± 7	n.s.
Recovery	Bulk shoot	421 ± 4	422 ± 4	n.s.
	Shoot sucrose	12 ± 0	13 ± 1	n.s.
	Shoot fructan	47 ± 4	33 ± 3	^∗∗^
	Shoot starch	9.0 ± 1.3	8.5 ± 0.8	n.s.
	Bulk root	357 ± 7	379 ± 8	^(∗)^
	Root sucrose	4.4 ± 0.6	2.7 ± 0.1	^∗∗∗^
	Root fructan	35 ± 6	29 ± 3	n.s.
	Root starch	21 ± 4	14 ± 4	n.s.


*^*a*^Levels of significance for drought effects: ^∗∗∗^P_*aovp*_ < 0.001, ^∗∗^P_*aovp*_ < 0.01, ^∗^P_*aovp*_ < 0.05, ^(∗)^P_*aovp*_ < 0.1; n.s., not significant. *Values represent averages among the mesocosms for each treatment (mean ± SE, n = 6), after averaging over the four sampling times for each mesocosm.**

**FIGURE 2:**
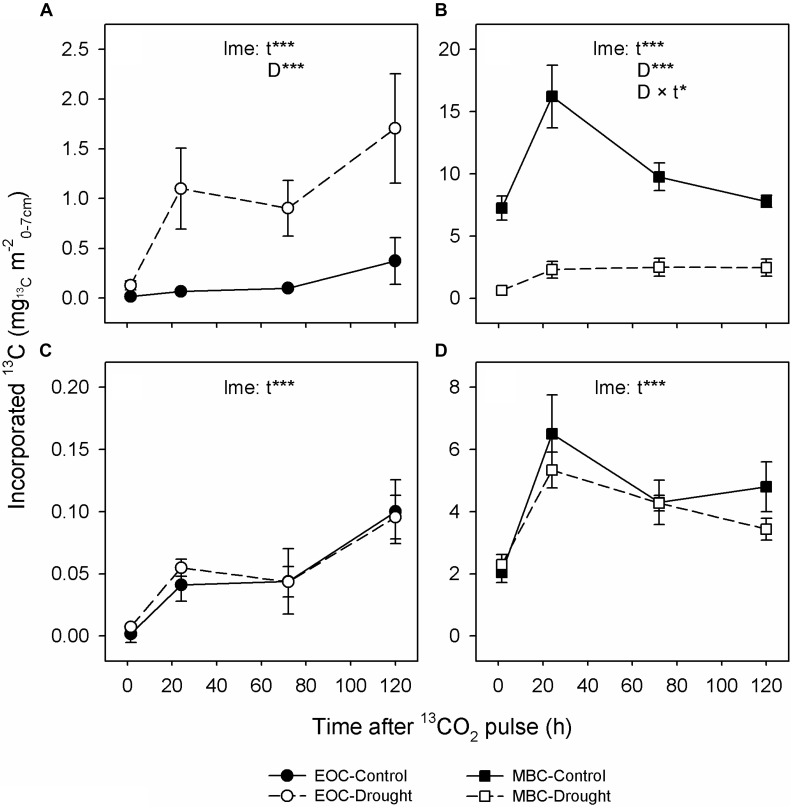
Dynamics of ^13^C tracer incorporation into extractable organic carbon (EOC; circles; **A,C**) and microbial biomass carbon (MBC; squares; **B,D**) from soil of control (closed symbols and solid lines) and drought-treated (open symbols and dashed lines) mesocosms at the peak drought **(A,B)** and recovery **(C,D)** labeling campaigns. Error bars show the SE of *n* = 6 mesocosms. Levels of significance for time after labeling (*t*; df = 3), drought treatment (D; df = 1) and the interaction of both (D × t; df = 3) were obtained from linear mixed-effects (lme) models using the R package ‘lme4’; ^∗∗∗^*P*_χ^2^_ < 0.001, ^∗∗^*P*_χ^2^_ < 0.01, ^∗^*P*_χ^2^_ < 0.05. Note that the labeling time was 30 min at the recovery labeling compared to 75 min at the peak drought labeling and that the absolute values cannot be compared between the labeling campaigns.

Regarding the soil, drought led to a threefold increase of water-soluble EOC compared to controls (Table 3) but had no effect on the MBC content. Significantly higher relative ^13^C allocation to the EOC (Figure 1A and Supplementary Figure S4) resulted from the continuous increase of ^13^C tracer incorporation into the EOC after the labeling (Figure 2A). By contrast, drought consistently reduced the amount of ^13^C tracer incorporation into MBC over time and delayed the label uptake (Figure 2B), leading to lower relative ^13^C allocation to MBC at 15 min and 24 h (Supplementary Figure S4). The reduced microbial ^13^C incorporation during drought was more pronounced for the individual lipid markers (Supplementary Figures S8A–D), yielding significantly decreased relative ^13^C allocation to AM fungi, saprotrophic fungi, and Gram-negative and Gram-positive bacteria (Figure 1A). This effect was not visible for actinobacteria (Figure 1A), which, on average, did not incorporate detectable amounts of ^13^C in control and drought treatments in their lipid markers (Supplementary Figure S8E). AM fungi, which took up the largest amount of ^13^C in the controls, reflected the tracer dynamics of MBC (Figure 2B and Supplementary S8A). This relation was less pronounced for saprotrophic fungi, whereas bacteria showed a slower label uptake. At the biomass scale, AM fungi were slightly affected by drought, whereas saprotrophic fungi were unaffected, and the bacterial biomass generally increased (Table 3).

**Table 3:** Effects of drought on the sizes of soil carbon and microbial marker lipid pools for the peak drought and the recovery labeling campaigns.

Labeling	Parameter	C content (μg_C_ g_dm_^-1^)		
		
		Control	Drought	*D* ^a^
Peak drought	EOC	34 ± 4	102 ± 8	^∗∗∗^
	MBC	402 ± 33	429 ± 20	n.s.
	AM fungi	24 ± 3	17 ± 2	^∗^
	Saprotrophic fungi	1.1 ± 0.1	1.2 ± 0.2	n.s.
	Gram (-) bacteria	5.7 ± 0.4	7.1 ± 0.3	^∗∗^
	Gram (+) bacteria	4.1 ± 0.3	4.8 ± 0.2	^∗^
	Actinobacteria	2.4 ± 0.2	2.9 ± 0.1	^∗^
Recovery	EOC	32 ± 3	32 ± 1	n.s.
	MBC	393 ± 18	393 ± 15	n.s.
	AM fungi	34 ± 2	19 ± 2	^∗∗∗^
	Saprotrophic fungi	0.9 ± 0.1	0.9 ± 0.1	n.s.
	Gram (-) bacteria	6.0 ± 0.4	6.6 ± 0.4	n.s.
	Gram (+) bacteria	4.3 ± 0.3	4.6 ± 0.4	n.s.
	Actinobacteria	2.8 ± 0.2	2.9 ± 0.2	n.s.


*^*a*^Levels of significance for drought effects: ^∗∗∗^P_*aovp*_ < 0.001, ^∗∗^P_*aovp*_ < 0.01, ^∗^P_*aovp*_ < 0.05, ^(∗)^P_*aovp*_ < 0.1; *n.s., not significant. AM, arbuscular mycorrhizal; EOC, extractable organic carbon; MBC microbial biomass carbon. Values represent averages among the mesocosms for each treatment (mean ± SE, n = 6), after averaging over the sampling times (four for EOC and MBC, two for microbial marker lipids) for each mesocosm.**

### Recovery Labeling

Ten days after rewetting, drought-treated mesocosms fully recovered their shoot biomass, root:shoot ratio, ^13^C uptake (Table 1), and photosynthetic rate (Supplementary Figure S5B). Accordingly, the amount of ^13^C incorporated in the root and shoot pools mostly recovered (Supplementary Figures S6I–P). NSC tracer dynamics partially differed between the control and drought treatments. Drought led to an earlier peak value of ^13^C incorporation into root sucrose (Supplementary Figure S6N) and to faster label decreases in shoot starch and root fructan after peak values were reached (Supplementary Figures S6L,O). This also resulted in a lower relative ^13^C allocation to root sucrose 72 h and 120 h after labeling (Supplementary Figure S4), whereas carbon allocation to shoot and root storage was only little affected. Bulk roots mainly reflected the ^13^C tracer dynamics of root fructan, showing a similar trend over time (Supplementary Figures S6M,O), i.e., a decrease of ^13^C incorporation at 72 h. Despite largely recovered carbon fluxes, the previous drought caused reductions in the concentrations of shoot fructan and root sucrose at the recovery labeling (Table 2). The overall root respiration rate was not affected by drought and rewetting (Table 1) but was increased at the dry mass level (Control, 4.6 ± 0.8 nmol_CO2_ g^-1^_dm_ s^-1^; Drought, 5.7 ± 0.6 nmol_CO2_ g^-1^_dm_ s^-1^; *P_aovp_* = 0.039). Furthermore, root respiration had similar ^13^C tracer dynamics like root sucrose, showing an earlier peak of respired ^13^C in drought-treated mesocosms (Supplementary Figure S7B). Rewetting led to significantly higher nitrogen concentrations in the roots (Control, 0.80 ± 0.05%_N_; Drought, 0.98 ± 0.05%_N_; *P_aovp_* = 0.006) and shoots (Control, 1.18 ± 0.05%_N_; Drought, 1.69 ± 0.11%_N_; *P_aovp_* < 0.001), thereby increasing the shoot and total biomass N content per unit area (Table 1).

Overall, plant and soil-related parameters recovered from drought at the recovery labeling. Consistently, the concentrations and ^13^C tracer incorporations of EOC and MBC fully recovered (Table 3 and Figures 2C,D). The ^13^C uptake in different microbial groups also recovered and showed little variation between the groups (Supplementary Figures S8F–J). Only the relative ^13^C allocation to saprotrophic fungi was significantly increased after rewetting (Figure 1B), as visible by the slightly higher ^13^C incorporation into the saprotrophic fungal marker (Supplementary Figure S8G). A similar trend was present for the tracer incorporation into Gram-negative bacterial markers, while no effect was observed on the Gram-positive bacterial markers. In contrast, for the AM fungal marker, a weak trend existed, showing a reduction in the ^13^C incorporation in drought mesocosms. This trend corresponded to a significantly reduced marker concentration (Table 3), which was largely counterbalanced by a higher relative abundance of ^13^C tracer (atom% ^13^C_excess_) (Supplementary Figure S9). For all other microbial groups, the marker concentrations were equal between control and drought treatments.

## Discussion

In a previous experiment on intact vegetation-soil monoliths from a managed meadow and an abandoned grassland, we found that drought-induced reductions of plant photosynthetic activity (Ingrisch et al., 2018) were coupled to strong reductions in plant storage NSCs, especially above ground, whereas BCA was maintained at a constant level (abandoned grassland) or even increased (managed meadow) relative to the total carbon uptake (Karlowsky et al., 2018). The carbon allocated to roots was largely recovered in drought-accumulated soluble sugars, whereas the uptake of plant-derived carbon in fatty acid biomarkers of root-associated microorganisms (AM fungi, SF and bacteria) was strongly reduced. Overall, these responses were greater in the managed meadow compared to the abandoned grassland, which likely also profited from enhanced AM fungal growth during drought. Furthermore, we found that after rewetting, the carbon uptake of the SF and bacteria was enhanced in the managed meadow (Karlowsky et al., 2018), which was reflected by higher plant nitrogen uptake and a faster recovery of aboveground biomass compared to the abandoned grassland (Ingrisch et al., 2018).

However, we were not able to assess whether the accumulation of root sugars during drought affected the release of carbon to the rhizosphere, nor were we able to determine how the drought-induced shift toward belowground allocation in the meadow might be related to its quick recovery after rewetting. Therefore, the aim of this study was to further elucidate the mechanisms underlying the link between plant photosynthesis and soil microbial carbon cycling during drought and after rewetting.

### The Link Between Plant and Soil Microbial Processes at Peak Drought

Surprisingly, drought had no significant effect on the total plant biomass. However, the decrease in shoot biomass and the concurrent increase in fine root biomass indicate that drought led to a shift in plant carbon allocation toward the belowground organs. Similar results have been found before in drought experiments on managed grasslands (Kahmen et al., 2005; Burri et al., 2014) and were attributed by the authors to an adaptation of plants in order to forage the limited water in dry soil. However, the root biomass response to drought can vary (Kahmen et al., 2005) and depends on the severity of the drought (Kreyling et al., 2008). Another root response occurring together with increased BCA is the accumulation of root sugars, especially sucrose (Hasibeder et al., 2015; Karlowsky et al., 2018). Such accumulations of root sugars can indicate an adjustment to dry conditions (Hasibeder et al., 2015) by increasing the concentration of osmolytes that prevent cells from desiccation (Chaves et al., 2003; Chen and Jiang, 2010). In our study, simultaneously increased concentrations of free glucose and fructose in roots (data not shown) further point to osmotic adjustment (Chen and Jiang, 2010).

**FIGURE 3:**
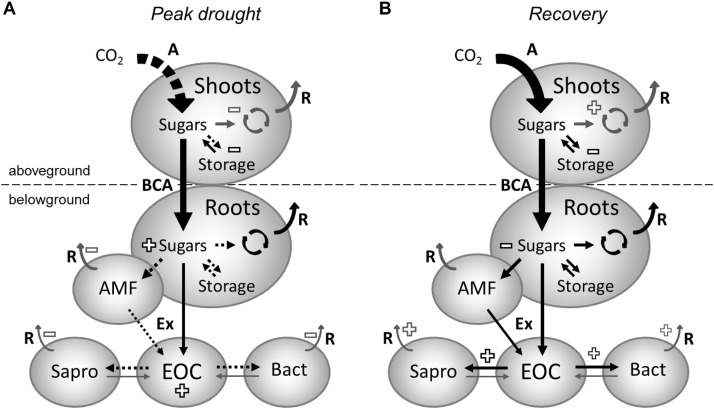
Effects of drought **(A)** and rewetting **(B)** on carbon fluxes and pools in grassland ecosystem. **(A)** During drought, assimilation **(A)** is reduced (reductions shown as dashed arrows). This leads to reduced carbon allocation to aboveground storage decreasing its pool size (effects on pool sizes shown as “+” or “–” signs). Presumably, carbon allocation to shoot growth, maintenance and respiration (R) is also reduced during drought (fluxes that were not determined in this study are represented by gray arrows). Belowground carbon allocation (BCA) is maintained during drought and leads to the accumulation of root sugars because carbon allocation to storage and mycorrhizal interactions are reduced. The size of the root storage pool is unaffected, as its activity is reduced during drought. Root sugars are partially used for root growth and maintenance. Furthermore, there is ongoing exudation (Ex) of new assimilates by roots but not by AM fungi (AMF), leading to an increase of the extractable organic carbon (EOC) in the soil, as the carbon uptake and metabolic activity of saprotrophic fungi (Sapro) and bacteria (Bact) is strongly reduced during drought. Shortly after rewetting **(B)** carbon assimilation and allocation mostly recovers. Because reductions still occur in the shoot storage pool, it is likely that priority is given to shoot re-growth. Accumulations of root sugars and EOC observed during drought rapidly vanish after rewetting and are likely used for priming soil microbial activity. In addition, the root sugar pool is reduced due to a faster carbon turnover, which is associated with increased transfer of newly assimilated carbon to saprotrophic fungi and (by tendency) bacteria in the rhizosphere, indirectly suggesting increased root/mycorrhizal exudation.

Independently of its usage, the carbon needed to maintain BCA originates either from recent assimilates or from remobilized aboveground storage compounds. In previous studies, drought increased the proportion of recently assimilated carbon allocated belowground (Palta and Gregory, 1997; Huang and Fu, 2000; Burri et al., 2014; Hasibeder et al., 2015; Karlowsky et al., 2018). Here, we could not identify this effect (Figure 3A), suggesting a higher contribution of shoot storage is needed to maintain BCA during drought, as indicated by the depletion of shoot fructan and starch. This might be due to stronger negative effects of drought on carbon assimilation than in the previous studies. Diverging results for the belowground allocation of freshly assimilated carbon have been reported before by Sanaullah et al. (2012) in a lab-based mesocosm experiment with monocultures and different mixtures of two grasses and one legume, whereas Ruehr et al. (2009) even found that drought increased the residence time of new carbon in leaves from beech trees. Of course, as woody species, trees have additional aboveground storage organs, which likely modify their drought response compared to herbaceous species. As a consequence, the source of the typically observed increase of BCA during drought might vary between fresh assimilates and older reserve carbohydrates, depending on the severity of drought, the timing in the year, as well as the functional composition or type of plants. In general, as previously concluded by Bahn et al. (2013), under reduced carbon supply, BCA in grassland seems to be maintained at the expense of aboveground storage (Figure 3A). Furthermore, the increase of nitrogen content in the roots (g_N_ m^-2^) of drought-treated plants (Table 1) suggests that the disturbance-adapted meadow plants actively preserve their resources belowground during extreme drought, likely to facilitate quick recovery after rewetting (Karlowsky et al., 2018).

Most interestingly, the altered plant resource allocation patterns did not disrupt the release of recently assimilated carbon to the rhizosphere during drought (Figure 3A), as visible by the high amount of ^13^C tracer in the soil EOC fraction, which exceeded control levels shortly after labeling. A similar enrichment of plant-derived carbon in the EOC pool was found by Fuchslueger et al. (2014a) and was attributed by the authors to the role of root exudates in reducing friction resistance in soil and maintaining root-soil connectivity. However, the strong reduction in ^13^C recovered in the microbial biomass of drought mesocosms points to decreased microbial uptake of recent plant-derived carbon, which probably led to the strong accumulation of carbon in the EOC pool. Nonetheless, increased root exudation during drought, as evidenced by a recent mesocosm study on tree saplings (Preece et al., 2018), could have further contributed to the greater EOC pools in the soil.

Notably, the relative ^13^C allocation to MBC was much less reduced by drought compared to microbial marker fatty acids (Figure 1A). This finding may imply that drought-reduced microbial growth, which can be estimated by the production of new fatty acids, and led to the accumulation of osmotically active compounds in MBC (Schimel et al., 2007). Osmolytes, e.g., amino acids in bacteria and polyols in fungi, are essentially highly water soluble and are more easily recovered than hydrophobic fatty acid-containing lipids in the MBC, which is extracted using aqueous K_2_SO_4_ solution. Moreover, reduced substrate diffusion, assumed to be the main limiting factor for bacterial activity in dry soil (Skopp et al., 1990; Stark and Firestone, 1995; Nunan et al., 2017), cannot explain the reduced ^13^C tracer uptake by AM fungi during drought, since mycorrhizal interactions do not depend on substrate diffusion in the soil.

Unexpectedly, bacterial biomass was generally higher in drought-treated mesocosms (Table 3). A high resistance to drought was expected for the slow-growing, Gram-positive (actino)bacteria but not for the Gram-negative bacteria with their thin cell wall (Schimel et al., 2007; Lennon et al., 2012). Possibly, Gram-negative bacteria profited from the increased root growth and exudate availability during drought, as the increased amounts of EOC in drought mesocosms at peak drought labeling suggested. If this scenario occurred at earlier stages of drought, when soil moisture conditions were not limiting the bacterial activity, then Gram-negative bacteria could have used the easily consumable carbon from the EOC pool for their growth. Similarly, we did not expect the concentration of AM fungi marker in drought mesocosms to be reduced compared to the controls (Table 3). This contrasts previous findings from grassland monoliths (Karlowsky et al., 2018), showing an increase of the (AM + saprotrophic) fungi:bacteria ratio at peak drought. This difference could be due to the use of sieved soil in mesocosms, because the mycorrhizal network strongly interacts with soil structure (Rillig and Mummey, 2006). Other explanations include increased competition for plant carbon between fine roots and AM fungi, or a lower plant dependence on AM fungi due to (a) lower nutrient demand of senescing shoots or (b) higher nutrient availability resulting from decreased competition with soil microorganisms. Additionally, the selected plant species might have interacted differently with AM fungal populations (Legay et al., 2016; Mariotte et al., 2017). Additionally, bacterial foraging of senescing AM fungi structures cannot be excluded and might have contributed to the increase in the Gram-negative bacteria during drought, too.

### Carbon Allocation and Plant–Microbial Interactions During Recovery

After rewetting, the mesocosm communities quickly recovered from drought, and both the shoot biomass and the root:shoot ratio were restored to control levels (Table 1). The higher fine root growth observed during drought was ceased at recovery labeling, possibly to support the re-growth of shoot biomass. However, the mechanisms behind the change in fine root biomass remain unclear, and thus, we cannot exclude the possibility that this observation was due to initial differences between the mesocosms used for the peak drought labeling and the mesocosms used for the recovery labeling. In general, the root response to drought-rewetting seems to be highly variable because previous studies either found an increase (Fuchslueger et al., 2016; Karlowsky et al., 2018, abandoned grassland) or no change (Karlowsky et al., 2018, managed meadow) in the fine root biomass after rewetting. In the latter study, the root response depended on the land use and was attributed to variable needs of water and nutrient uptake by fine roots, resulting from differences in the recovery of the dominant plant-microbial interactions. On the other side, in this study, the plant ^13^C tracer uptake and allocation supports the hypothesis that carbon resources are preferentially invested into the regrowth of shoot biomass after rewetting (Figure 3B). Despite recovered ^13^C tracer dynamics, the reduced shoot fructan pool indicates that, during the recovery phase, plants invested more carbon into structural carbohydrates or into respiration (e.g., for repair processes) than in storage. This investment was underpinned by the higher turnover of ^13^C tracer in shoot starch, which suggests a faster utilization of recent assimilates from transitory storage (Bahn et al., 2013) in plants recovering from drought. The reduced concentrations of root sucrose after rewetting could also be a result of the preferential use of newly assimilated carbon for shoot regrowth, decreasing the BCA during recovery (Zang et al., 2014). However, since only a marginal effect was observed on the average ^13^C tracer incorporation in root sucrose and apparently a faster utilization of recent assimilates occurred in roots (Supplementary Figures S6M–O), most likely, the reduced sucrose concentrations were a result of increased root-rhizosphere carbon transfer (Hagedorn et al., 2016).

According to a shift in root functioning from resource preservation to nutrient acquisition, the uptake of fresh plant-derived carbon completely recovered for all microbial groups, and the carbon transfer to saprotrophic fungi even increased in the drought mesocosms (Figure 3B). These microorganisms were also found to rapidly take up recent plant-derived carbon in grasslands (de Deyn et al., 2011). In contrast to a previous study on the meadow (Karlowsky et al., 2018), we could not find significant excess uptake of ^13^C tracer in bacteria. However, we cannot exclude that the use of sieved subsoil in this study led to altered microbial responses compared to the undisturbed topsoil in the previous study, as the initial microbial community and its functioning might have differed. Moreover, the rapid uptake of plant-derived carbon by saprotrophic fungi agrees with a recently introduced framework for carbon flow in the rhizosphere by Ballhausen and de Boer (2016), who proposed that a large fraction of the labile carbon from root exudation is primarily taken up by saprotrophic fungi prior to its consumption by fungus-feeding bacteria. As expected, AM fungi generally took up the largest fraction of plant-derived carbon in the soil microbial community (Drigo et al., 2010; Mellado-Vázquez et al., 2016; Karlowsky et al., 2018) but recovered slowly after rewetting the dried soil (de Vries et al., 2012; Meisner et al., 2013; Karlowsky et al., 2018). Interestingly, despite their lower abundance, AM fungi completely recovered their ^13^C tracer uptake in drought treatments at the recovery labeling, suggesting that the efficiency of plant-mycorrhizal carbon flow increased at this time to support the recovery of the hyphal network.

The recovery of soil microbial growth after drought is typically preceded by a pulse of soil respiration directly after rewetting (Birch, 1958). However, those sources other than the released microbial osmolytes that contribute to the Birch effect are not well known, especially in planted soils (Canarini et al., 2017). Here, we found accumulations of carbon in the root sugar and soil EOC pools during drought, which quickly disappeared after rewetting. This strongly suggests that the release of these easy degradable carbon sources after the end of drought contributes to the acceleration of the soil microbial activity. Data not yet published on soil respiration from the ^13^C pulse labeling experiment described by Karlowsky et al. (2018) indicate that carbon assimilated during drought contributes to the Birch effect, as ^13^C applied to the monoliths during peak drought could be recovered in the soil respiration pulse after rewetting. Consequently, this means that the plant-derived carbon, which cannot be used by soil microorganisms during drought, is available for priming the microbial organic matter cycle in soil after rewetting. Such priming effects, e.g., observed after amending soil samples with fresh plant litter (Thiessen et al., 2013), are well-known to support plant growth by increasing nutrient mineralization from soil organic matter. An increase in nitrogen mineralization especially has been reported after rewetting dried soils (Borken and Matzner, 2009; Canarini and Dijkstra, 2015), and this increase probably contributed to the increased root and shoot nitrogen concentrations found at the recovery in this study. Additionally, the transport of preserved nitrogen from roots to shoots could have led to the significantly increased shoot nitrogen concentrations in drought treatments. As the leaf nitrogen concentration typically correlates with the photosynthetic activity (Wright et al., 2001; Milcu et al., 2014), the increased nitrogen uptake likely facilitated the higher assimilation rates needed for recovery (Ingrisch et al., 2018).

## Conclusion

The results from this study confirm our first hypothesis that the frequently observed weakening of the link between plant photosynthesis and soil microbial carbon cycling during drought is due to reduced microbial uptake rather than to reduced root exudation. Our data from the ^13^C pulse labeling experiments clearly show that recently assimilated plant carbon accumulates in the rhizosphere in the form of EOC during drought and that this accumulation is linked to reduced microbial uptake of plant-derived carbon. When the soil dries out, the limited diffusion leads to lower accessibility of root exudates for non-mycorrhizal fungi and bacteria. In addition, higher reductions of ^13^C tracer allocation to growth-related fatty acid markers in comparison to the water-soluble MBC fraction, also in AM fungi, indicate adjustments in microbial metabolic activity; that is, the formation of osmolytes to prevent cell desiccation is favored over growth.

Our second hypothesis that drought leads to the accumulation of root sugars and EOC and that these easy degradable carbon sources are available for priming plant and soil microbial activity after rewetting, is only partially supported by the data. Indeed, we found that carbohydrates accumulated in roots and that the decreased microbial uptake was linked to increased EOC concentrations during drought. However, what causes the depletion of drought-accumulated carbon after rewetting remains unclear. Root sugars could either be used to support the regrowth of shoots or may be invested in plant-microbial interactions to gain more nutrients from soil organic matter decomposition. Drought-accumulated EOC that is not flushed away during the rewetting potentially further fuels the Birch effect, i.e., high microbial carbon and nitrogen mineralization shortly after rewetting. To determine how the preservation of belowground carbon pools during drought is related to microbial activity in the early phase of ecosystem recovery, future studies are needed to trace the flux of ^13^C label applied at drought in soil after rewetting.

Ultimately, our results indicate that the link between plants and soil microorganisms plays a crucial role in the short-term response of carbon and nitrogen cycling to drought-rewetting events.

## Data Accessibility

The datasets analyzed for this study can be found in the figshare repository: https://figshare.com/s/afd9c8f0fab5a572fdb3.

## Author Contributions

MB and GG conceived the ideas. SK, AA, JI, MB, and GG designed the methodology. SK, AA, JI, MA, and GG conducted the experiments and collected the data. SK, AA, and MA analyzed the data. SK and GG led the writing of the manuscript. All authors contributed critically to the drafts and gave final approval for publication.

## Conflict of Interest Statement

The authors declare that the research was conducted in the absence of any commercial or financial relationships that could be construed as a potential conflict of interest.
