# Supplementary material for: Drought-Induced Accumulation of Root Exudates Supports Post-drought Recovery of Microbes in Mountain Grassland
Source: Front Plant Sci. 2018 Nov 7;9:1593. doi: 10.3389/fpls.2018.01593 (PMC6234839; doi:10.3389/fpls.2018.01593)
Supplement: Supplementary file 1 [file Table_1.docx]

Supplementary Material

Drought-Induced Accumulation of Root Exudates Supports Post-drought Recovery of Microbes in Mountain Grassland

Karlowsky S, Augusti A, Ingrisch J, Akanda MKU, Bahn M and Gleixner G*

*** Correspondence:** Corresponding Author: gerd.gleixner@bgc-jena.mpg.de

**Supplementary Table S1.** Planting schemata of mesocosms, with each scheme replicated in six mesocosms.

| **Species** | **Scheme 1** | **Scheme 2** | **Scheme 3** | **Scheme 4** |
| --- | --- | --- | --- | --- |
| *Deschampsia cespitosa* | 6 | 15 | 2 | 5 |
| *Festuca rubra* | 7 | 7 | 4 | 4 |
| *Dactylis glomerata* | 12 | 3 | 3 | 1 |
| *Leontodon hispidus* | 2 | 5 | 5 | 18 |
| *Geranium sylvaticum* | 5 | 2 | 18 | 4 |
| *Trifolium repens* | 4 | 4 | 4 | 4 |

**Supplementary Table S2.** Concentrations and 13C isotope content of CO2 in the labeling chamber measured on CO2 stable isotope analyzer (Picarro G2201i Analyzer, Picarro Inc., Santa Clara, CA, USA).

| **Labeling** | **Mesocosm** | ***n*^a^** |  | **CO_2_ concentration** | | | |  | **atom% ^13^C** | | | |
| --- | --- | --- | --- | --- | --- | --- | --- | --- | --- | --- | --- | --- |
|  |  |  |  | **min** | **max** | **mean** | **SD** |  | **min** | **max** | **mean** | **SD** |
| Peak drought | R31.M1 | 44 |  | 470 | 896 | 648 | 117 |  | 31 | 64 | 44 | 10 |
|  | R31.M5 | 47 |  | 320 | 927 | 580 | 151 |  | 56 | 83 | 69 | 8 |
|  | R32.M2 | 51 |  | 453 | 721 | 569 | 70 |  | 29 | 64 | 45 | 8 |
|  | R32.M4 | 34 |  | 405 | 802 | 613 | 95 |  | 53 | 77 | 64 | 7 |
|  | R33.M6 | 52 |  | 397 | 755 | 566 | 106 |  | 44 | 72 | 60 | 7 |
|  | R33.M7 | 61 |  | 337 | 704 | 516 | 89 |  | 41 | 75 | 61 | 10 |
|  | R34.M1 | 40 |  | 355 | 750 | 538 | 109 |  | 47 | 80 | 65 | 10 |
|  | R34.M7 | 40 |  | 448 | 730 | 584 | 83 |  | 36 | 60 | 49 | 7 |
|  | R35.M6 | 46 |  | 471 | 700 | 600 | 63 |  | 30 | 57 | 43 | 6 |
|  | R35.M7 | 41 |  | 355 | 751 | 522 | 112 |  | 30 | 64 | 46 | 9 |
|  | R36.M4 | 42 |  | 400 | 727 | 530 | 88 |  | 45 | 72 | 60 | 7 |
|  | R36.M6 | 46 |  | 372 | 735 | 548 | 105 |  | 22 | 70 | 42 | 14 |
| Recovery | R31.M2 | 45 |  | 520 | 802 | 654 | 74 |  | 59 | 77 | 70 | 5 |
|  | R31.M3 | 32 |  | 546 | 828 | 691 | 77 |  | 59 | 77 | 67 | 4 |
|  | R32.M3 | 25 |  | 465 | 776 | 611 | 92 |  | 50 | 70 | 59 | 7 |
|  | R32.M5 | 30 |  | 327 | 744 | 565 | 105 |  | 46 | 79 | 66 | 8 |
|  | R33.M2 | 32 |  | 484 | 774 | 604 | 75 |  | 44 | 64 | 54 | 6 |
|  | R33.M8 | 34 |  | 530 | 1046 | 752 | 144 |  | 55 | 78 | 62 | 6 |
|  | R34.M5 | 10 |  | 607 | 812 | 696 | 62 |  | 59 | 67 | 62 | 2 |
|  | R34.M8 | 10 |  | 629 | 822 | 729 | 74 |  | 60 | 68 | 64 | 3 |
|  | R35.M1 | 28 |  | 456 | 760 | 602 | 81 |  | 48 | 69 | 59 | 6 |
|  | R35.M4 | 19 |  | 544 | 1071 | 801 | 170 |  | 57 | 76 | 68 | 6 |
|  | R36.M1 | 28 |  | 532 | 910 | 677 | 129 |  | 55 | 74 | 64 | 6 |
|  | R36.M8 | 20 |  | 548 | 862 | 668 | 88 |  | 55 | 72 | 63 | 5 |

^a^*n* is the number of sufficient quality measurement points during the labeling period.


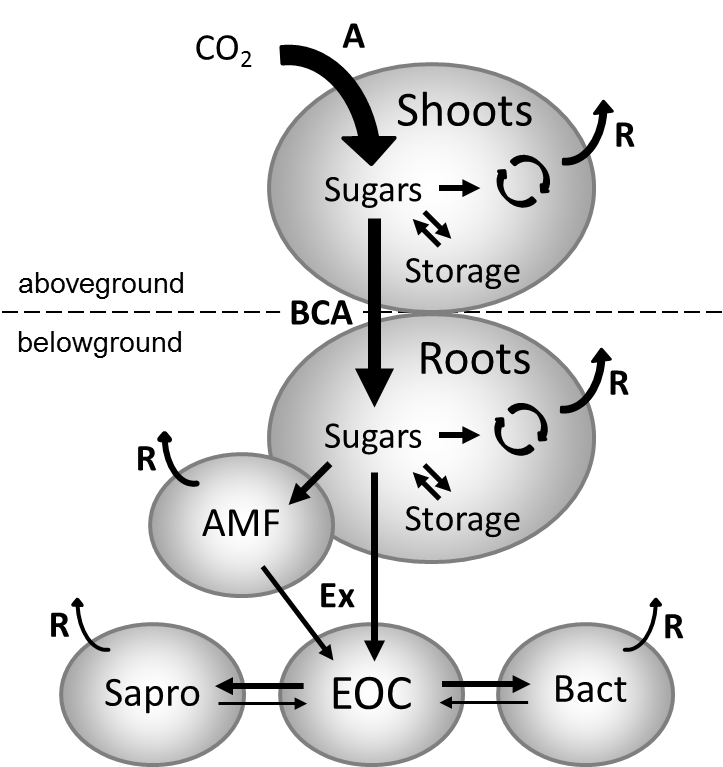


**Supplementary Figure S1.** Hypothetical pathway of newly assimilated carbon in the plant-rhizosphere system of grasslands (simplified). New carbon from assimilation (A) in shoots is either transformed for storage, used for growth and maintenance, associated with losses by respiration (R), or allocated to roots (belowground carbon allocation, BCA). Similarly, carbon in roots is either stored or used or further transferred to the rhizosphere and its inhabiting microorganisms. Carbon transfer to microbial biomass is possible directly through mycorrhizal interactions with arbuscular mycorrhizal fungi (AMF) or indirectly through exudation (Ex) into the rhizosphere/hyphosphere and uptake by saprotrophic fungi (Sapro) or bacteria (Bact). The extractable organic carbon (EOC) represents an intermediate pool, including exuded compounds as well as residues from dead cells, which can be accessed by saprotrophic fungi and soil bacteria.


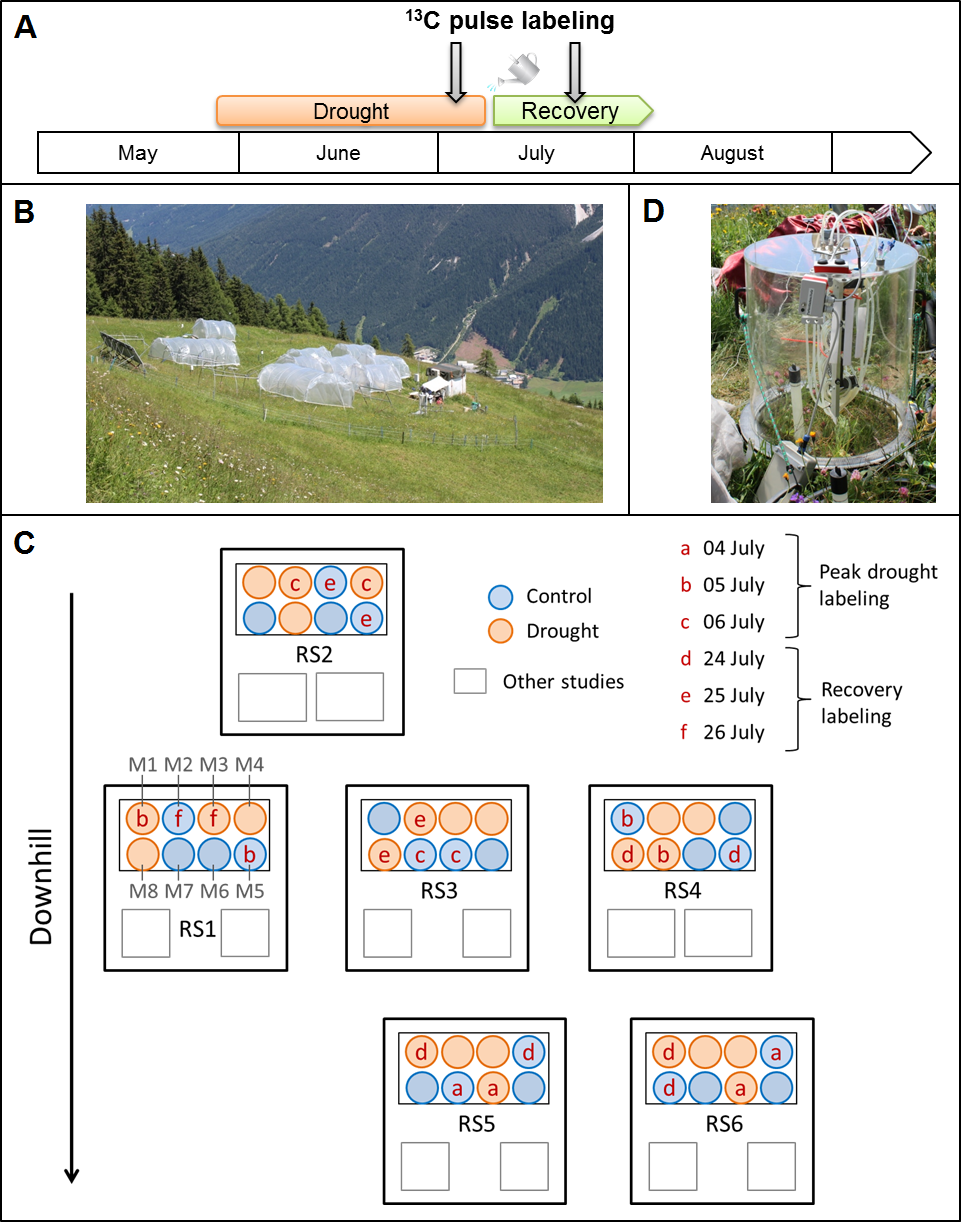


**Supplementary Figure S2.** Experimental timeline indicating the two ^13^C labeling campaigns at peak drought and recovery **(A)**; photography of the study site with transparent rainout shelters and a climate chamber used for operating the Picarro ^13^CO_2_ analyzer **(B)**; scheme of rain-out shelters (RS1-RS6) and mesocosms (M1-M8, see example for RS1), indicated are treatment (blue, control; orange, drought) and labeling dates **(C)**; and plexiglass labeling chamber on top of a mesocosm, sealed by a gas-tight rubber gasket, and cooled with ice water tubes coiled around several fans **(D)**.


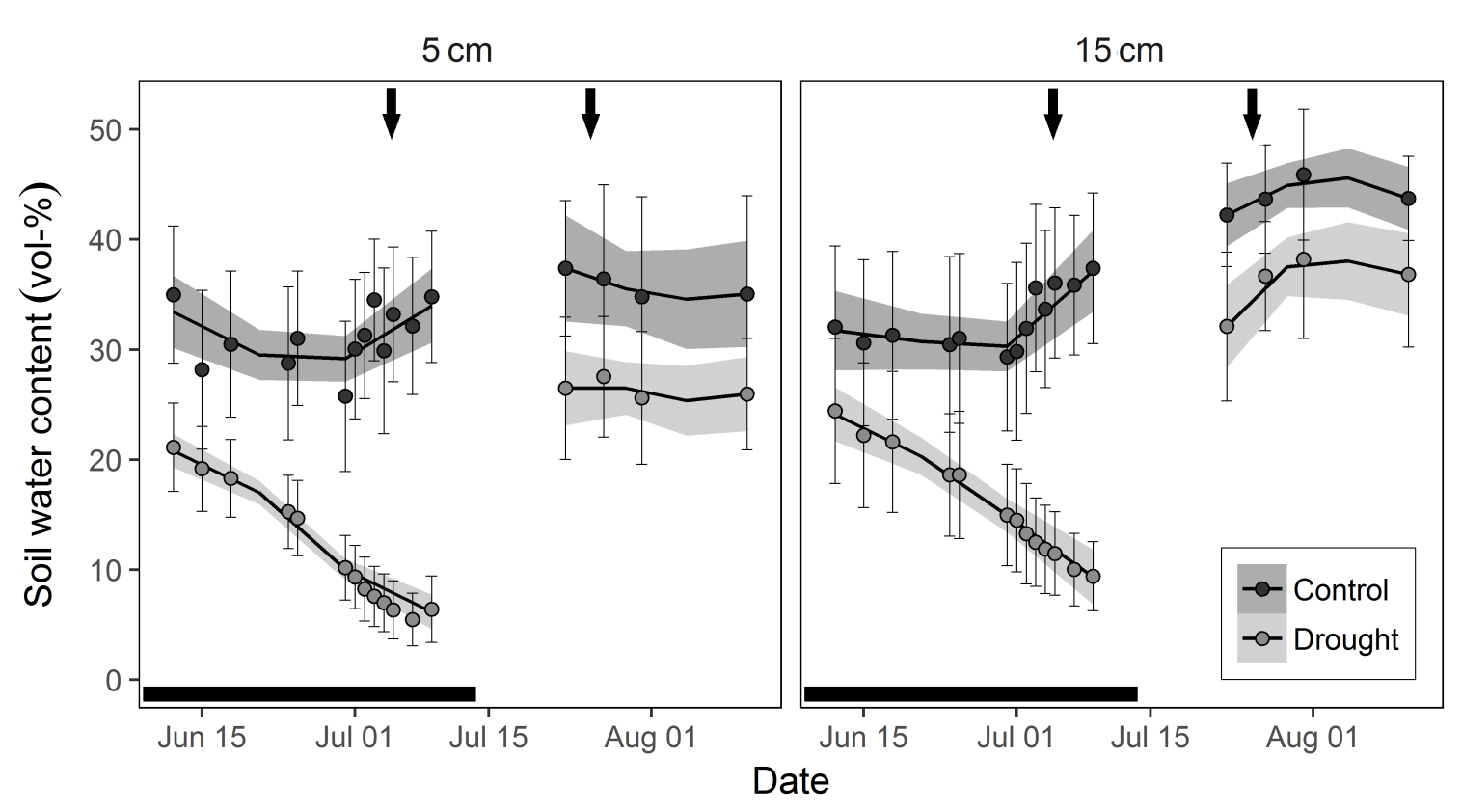


**Supplementary Figure S3.** Volumetric soil water content at soil depths of 5 cm (left) and 15 cm (right). The black bar above the horizontal axes indicates the time of drought simulation and arrows denote the dates of the two ^13^C pulse labeling campaigns. Circles show mean values ± SD (n = 12) of control (dark gray) and drought (light gray) treatments. Black lines show the results from local polynomial regression fitting (‘loess’ function from the R base package, evaluated at n = 4 data points) for each treatment and gray areas the corresponding 95% confidence intervals (control, dark gray; drought, light gray); breaks result from the rewetting event leading to sudden increases in soil water content, which cannot be reflected by the polynomial line fitting.


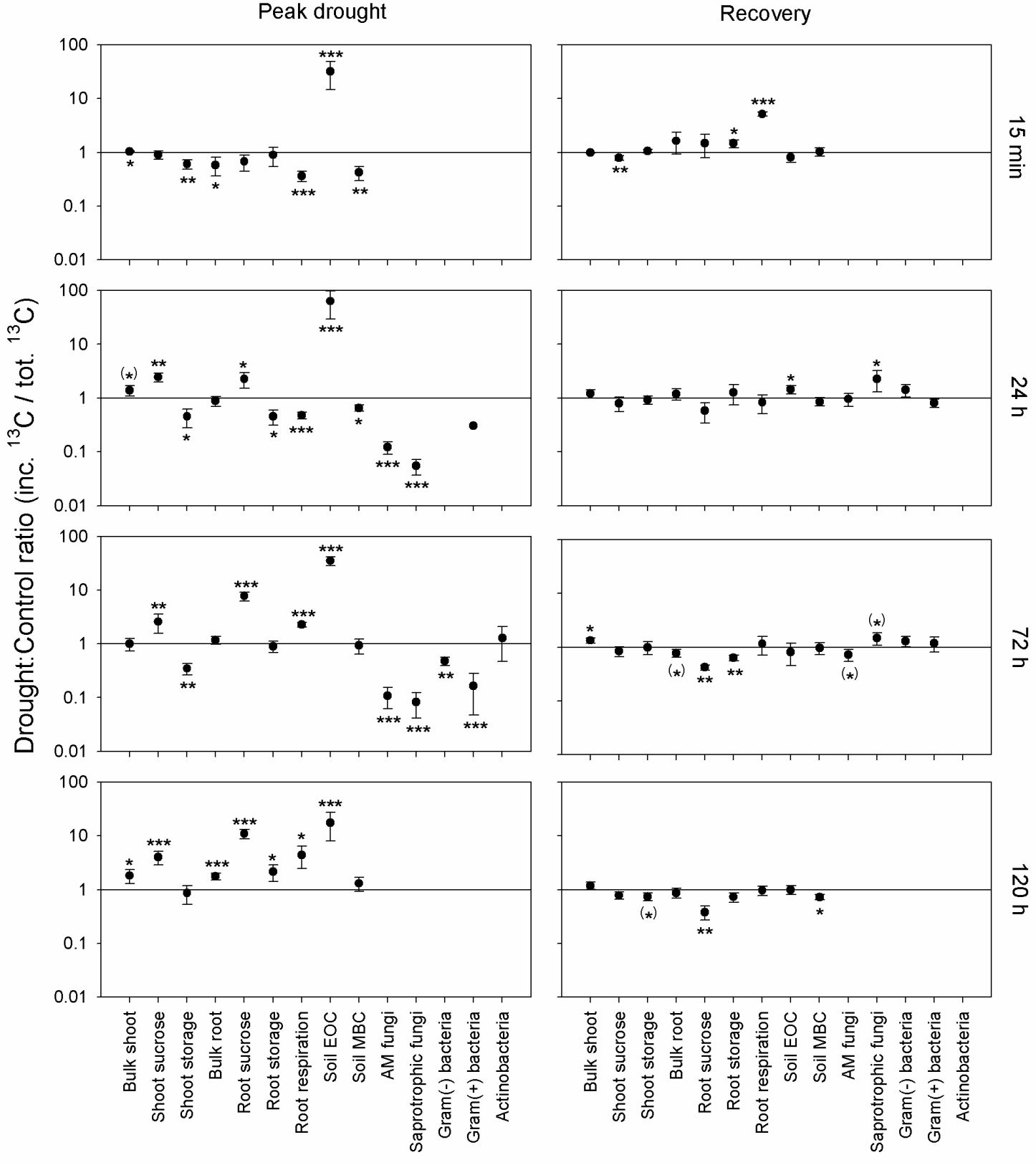


**Supplementary Figure S4.** Effects of drought on C allocation patterns at the peak drought and labeling campaigns. Shown is the drought to control ratio of the ^13^C amount in each pool that was recovered from total ^13^C uptake (relative ^13^C allocation) at the four different sampling times after pulse labeling. Black circles represent the mean of n = 1 to n = 6 control/drought pairs. Error bars were obtained by propagating the SE from the replicates of each treatment, control and drought respectively. Asterisks indicate significance levels of drought effects (df = 1) from linear mixed-effects models (R package ‘lme4’) using treatment as fixed factor and labeling pair and individual mesocosm as random factors; ****P_χ²_* < 0.001, ***P_χ²_* < 0.01, **P_χ²_* < 0.05, ^(^*^)^*P_χ²_* < 0.1.


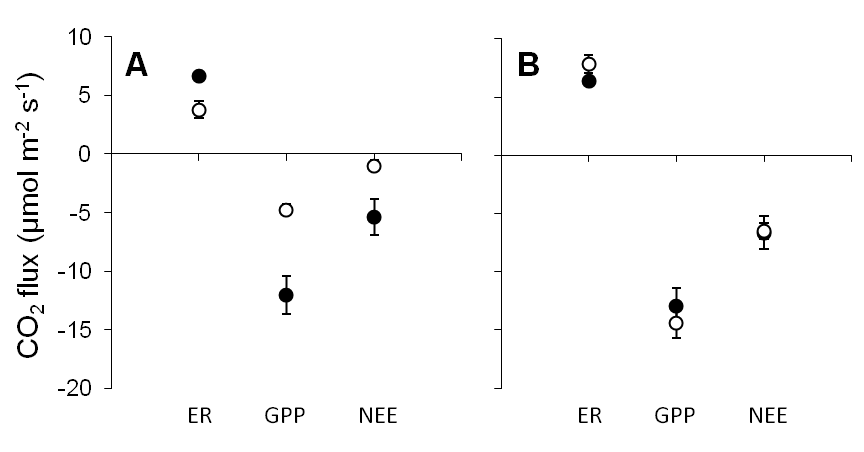


**Supplementary Figure S5.** Photosynthetic and respiration rates of control (closed circles) and drought (open circles) mesocosms at the peak drought **(A)** and recovery **(B)** labeling campaigns. Error bars represent SE of n = 6 (control mesocosms, peak drought labeling), n = 5 (drought mesocosms, peak drought labeling) or n = 4 (control and drought mesocosms, recovery labeling) mesocosms. Measurements of ecosystem respiration (ER) and net ecosystem exchange (NEE) were done by analyzing changes of chamber CO_2_ concentrations in the labeling chamber, under light (NEE) and dark (ER) conditions for a period of 1 minute each, on infrared gas analyzer (Licor 840A, Lincoln, NE, USA). Gross primary productivity (GPP), i.e., the photosynthetic rate, was calculated as: GPP = NEE − ER.


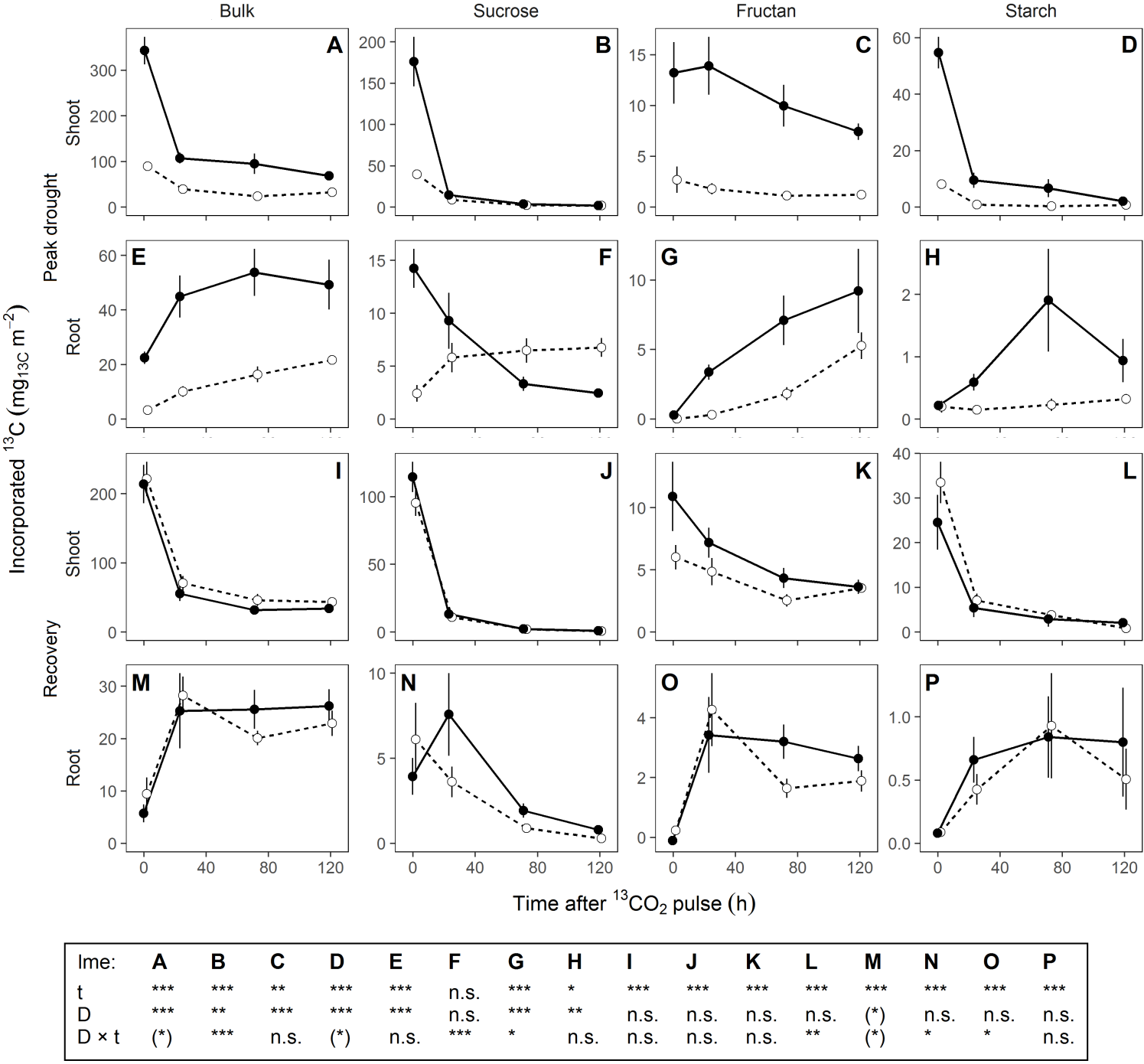


**Supplementary Figure S6.** Dynamics of ^13^C tracer incorporation into bulk shoots and roots as well as their carbohydrates from control (closed circles, solid lines) and drought (open circles, dashed lines) treatments at the peak drought **(A-H)** and the recovery **(I-P)** labeling campaigns. Error bars represent an SE of n = 6 (n = 5 for shoot starch, recovery, drought, 72 h). Levels of significance for time after labeling (t; df = 3), drought treatment (D; df = 1) and the interaction of both (D × t; df = 3) were obtained from linear mixed-effects (lme) models using the R package ‘lme4’; ****P_χ²_* < 0.001, ***P_χ²_* < 0.01, **P_χ²_* < 0.05 and (*)*P_χ²_* < 0.1. Note that the labeling time was only 30 min at the recovery labeling compared to 75 min at the peak drought labeling and that the absolute values cannot be compared between both labeling campaigns.


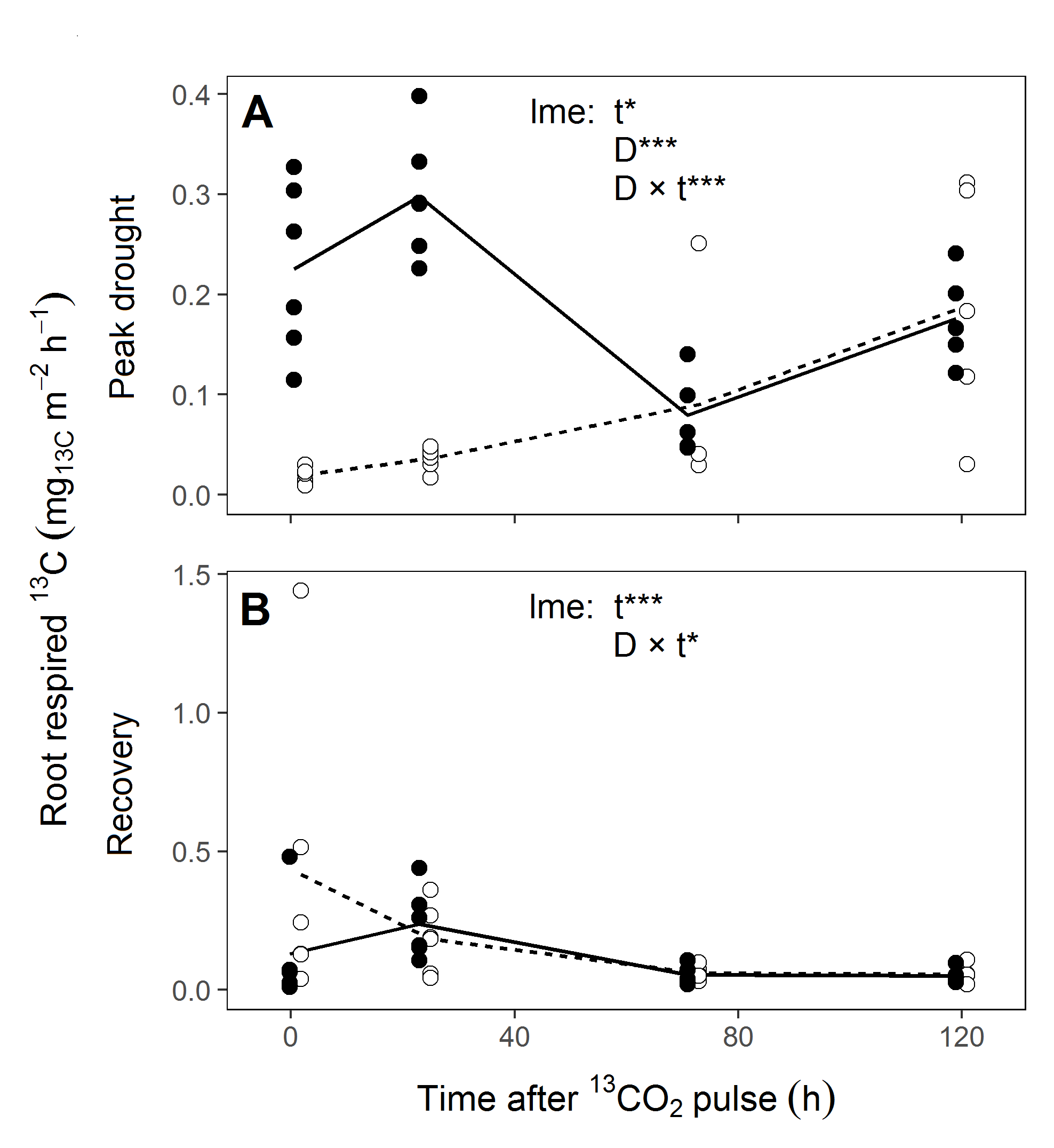


**Supplementary Figure S7.** Dynamics of ^13^C tracer incorporation into root respired CO_2_ at 15 °C from control (closed circles, solid lines) and drought (open circles, dashed lines) treatments at the peak drought **(A)** and the recovery **(B)** labeling campaigns. Circles show single values for each mesocosm and lines the mean values of n = 4-6 mesocosms at each sampling time. Levels of significance for time after labeling (t; df = 3), drought treatment (D; df = 1) and the interaction of both (D × t; df = 3) were obtained from linear mixed-effects (lme) models using the R package ‘lme4’; ****P_χ²_* < 0.001 and **P_χ²_* < 0.05. Note that the labeling time was only 30 min at the recovery labeling compared to 75 min at the peak drought labeling and that the absolute values cannot be compared between both labeling campaigns.


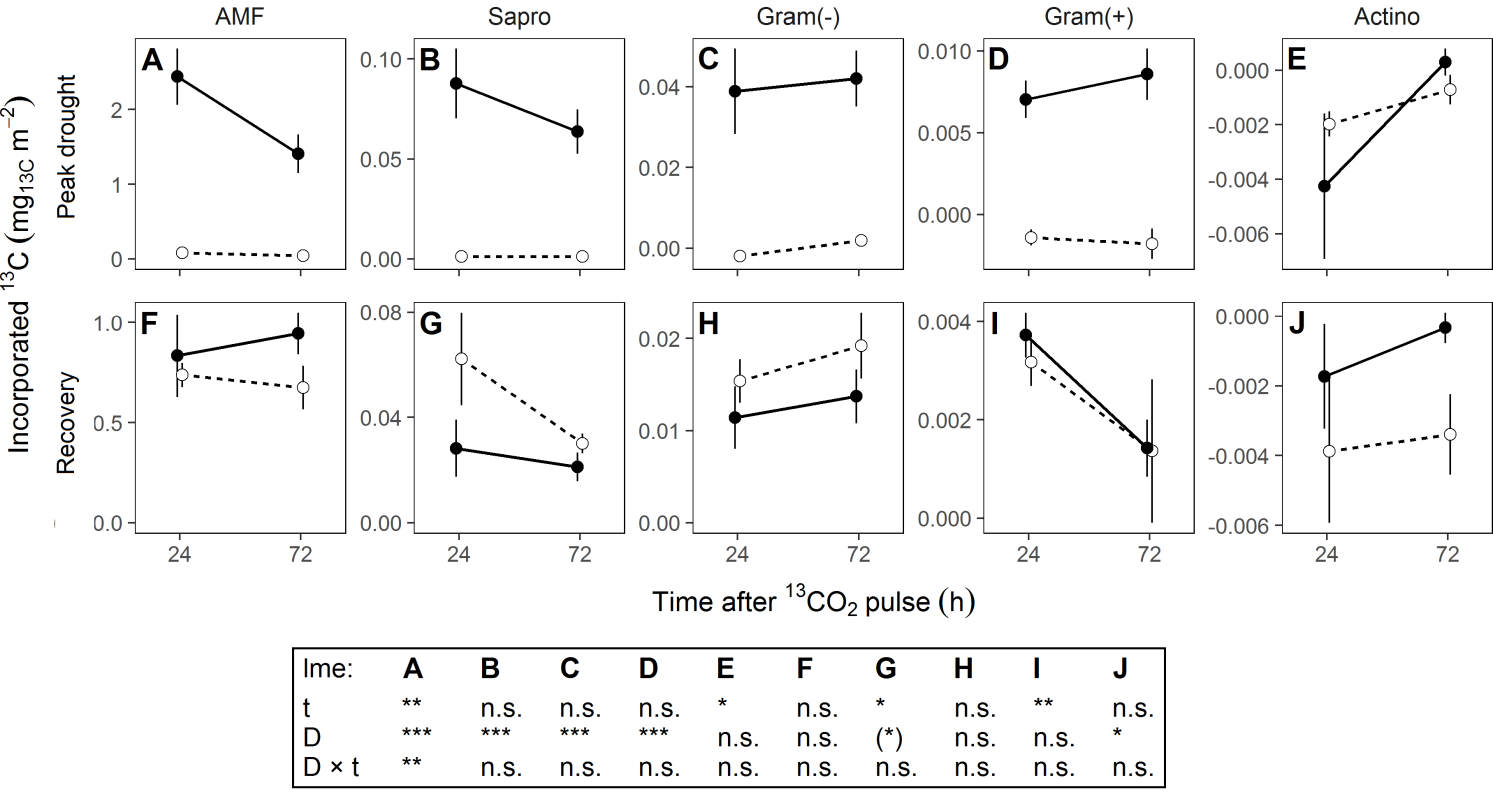


**Supplementary Figure S8.** Dynamics of ^13^C tracer incorporation into microbial marker lipids from soil of control (closed circles, solid lines) and drought (open circles, dashed lines) mesocosms at the peak drought **(A-E)** and recovery **(F-J)** labeling campaigns. Error bars represent SE of n = 6. Levels of significance for time after labeling (t; df = 3), drought treatment (D; df = 1) and the interaction of both (D × t; df = 3) were obtained from linear mixed-effects (lme) models using the R package ‘lme4’; ****P_χ²_* < 0.001, ***P_χ²_* < 0.01, **P_χ²_* < 0.05 and (*)*P_χ²_* < 0.1. Note that the labeling time was only 30 min at the recovery labeling compared to 75 min at the peak drought labeling and that the absolute values cannot be compared between the labeling campaigns. Negative incorporated ^13^C values result from negligible ^13^C tracer uptake and natural variations in ^13^C content between labeled and unlabeled reference samples. Actino, actinobacteria; AMF, arbuscular mycorrhizal fungi; Sapro, saprotrophic fungi; Gram(−)/(+) = Gram-negative/positive bacteria.


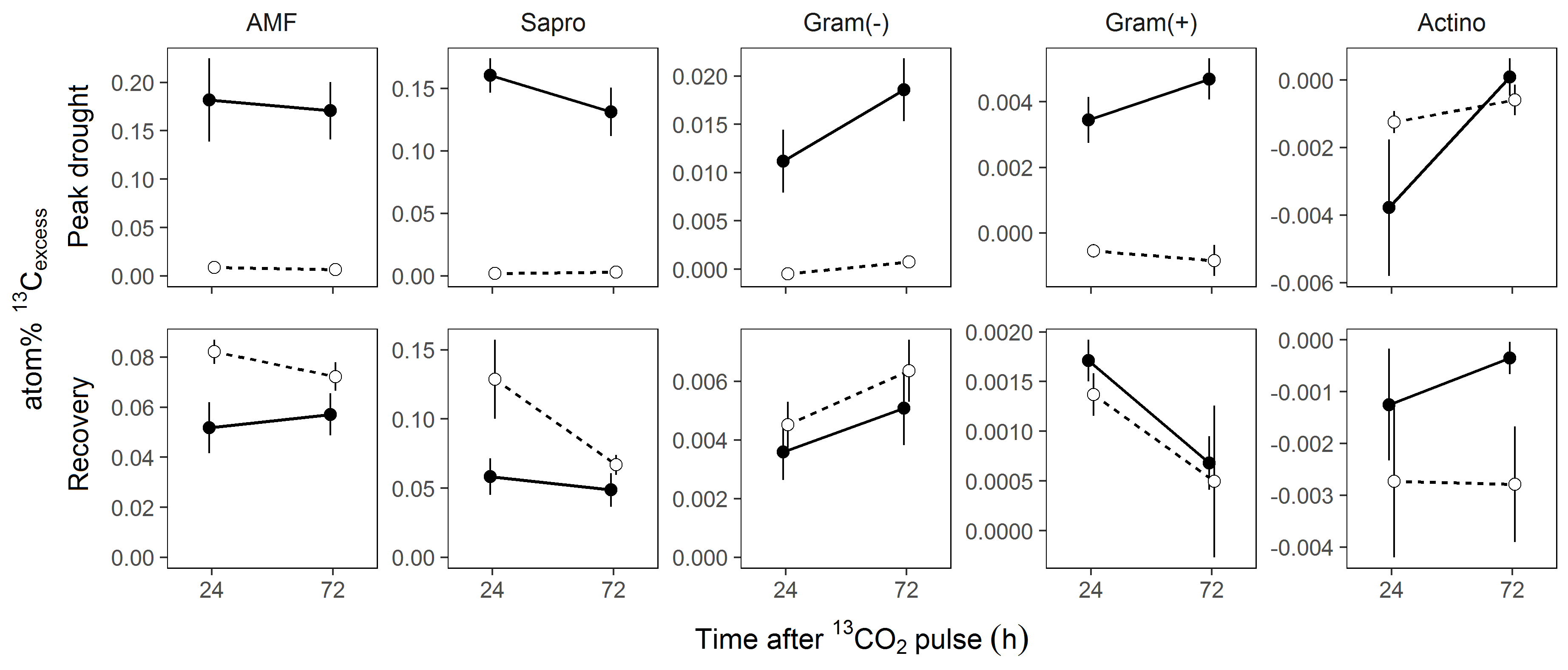


**Supplementary Figure S9.** Dynamics of atom% ^13^C_excess_ in microbial marker lipids from soil of control (closed circles, solid lines) and drought (open circles, dashed lines) mesocosms at the peak drought and recovery labeling campaigns. AMF: arbuscular mycorrhizal fungi, Sapro: saprotrophic fungi, Gram(−)/(+): Gram-negative/positive bacteria.


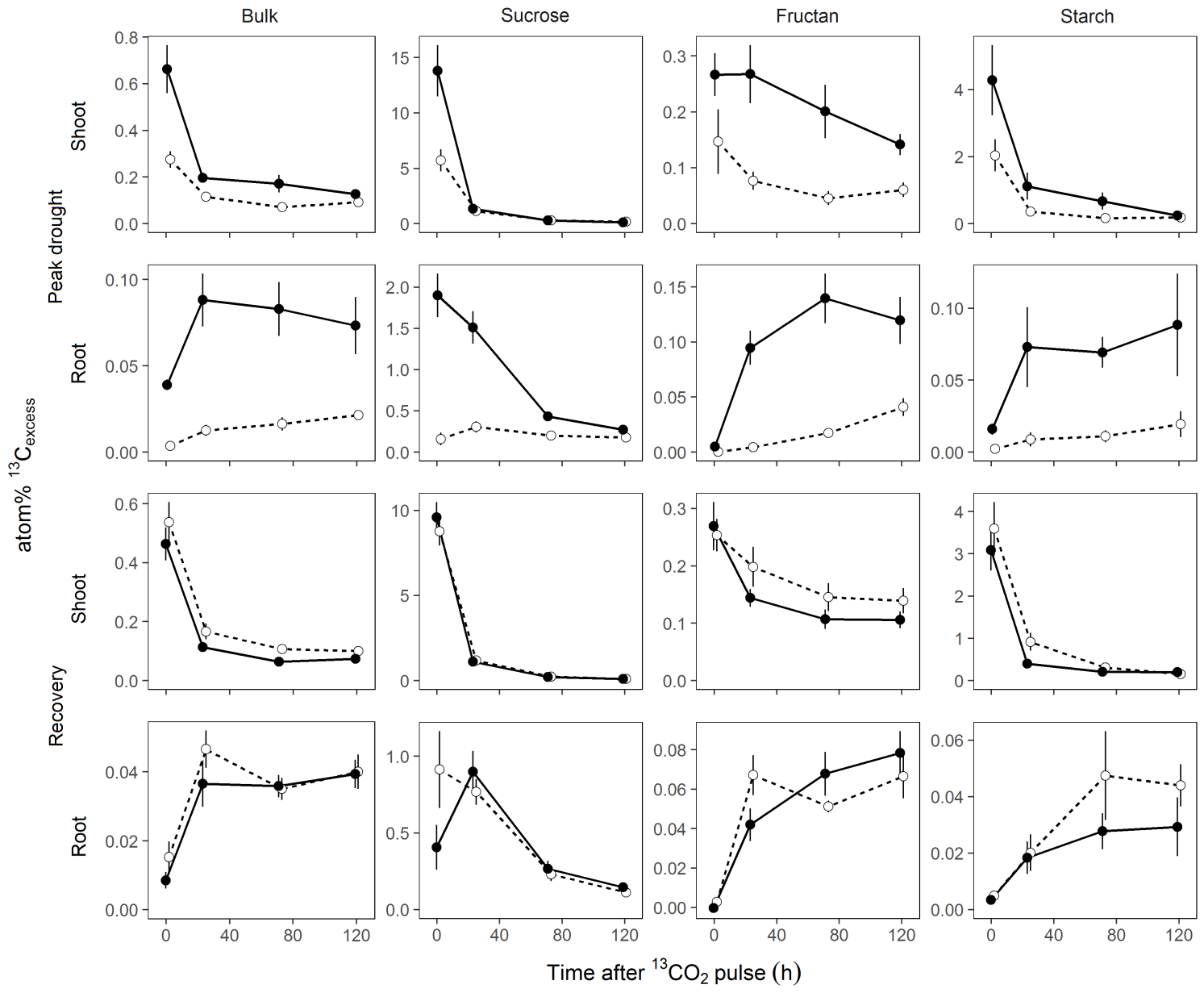


**Supplementary Figure S10.** Dynamics of atom% 13Cexcess in bulk shoots and roots as well as their carbohydrates from control (closed circles, solid lines) and drought (open circles, dashed lines) treatments at the peak drought and the recovery labeling campaigns. Error bars represent SE of n = 6 (n = 5 for shoot starch, recovery, drought, 72 h).


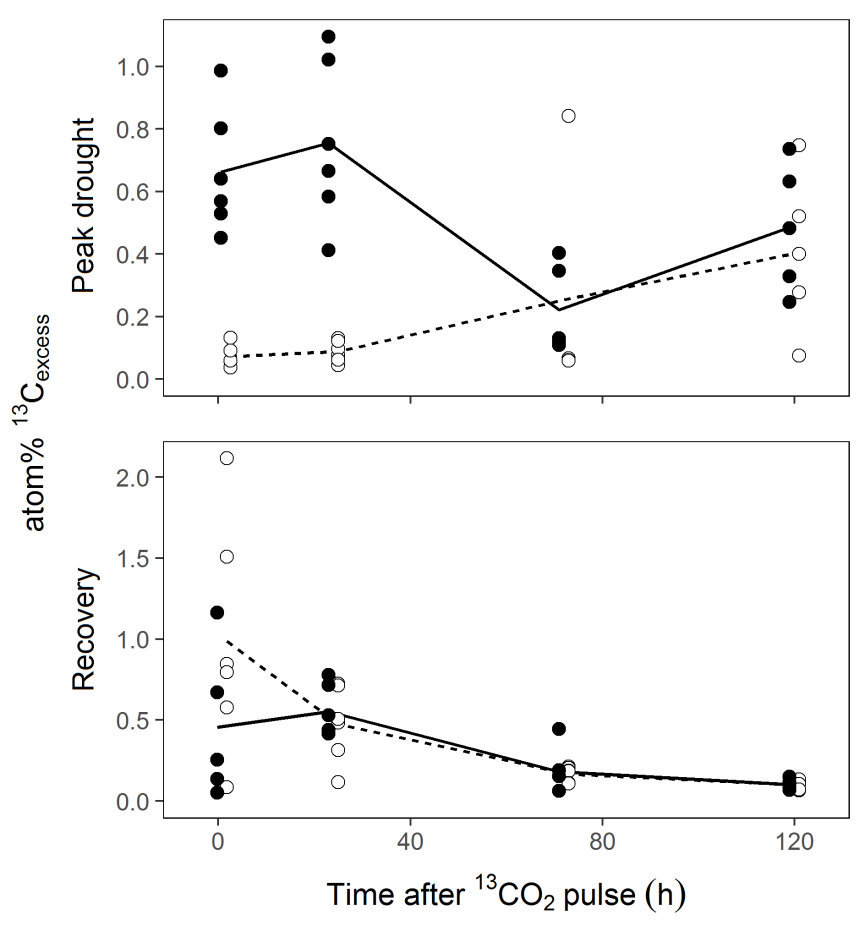


**Supplementary Figure S11.** Dynamics of atom% ^13^C_excess_ in root respired CO_2_ at 15 °C from control (closed circles, solid lines) and drought (open circles, dashed lines) treatments at the peak drought and the recovery labeling campaigns. Circles show single values for each mesocosm and lines the mean values of n = 4-6 mesocosms at each sampling time.


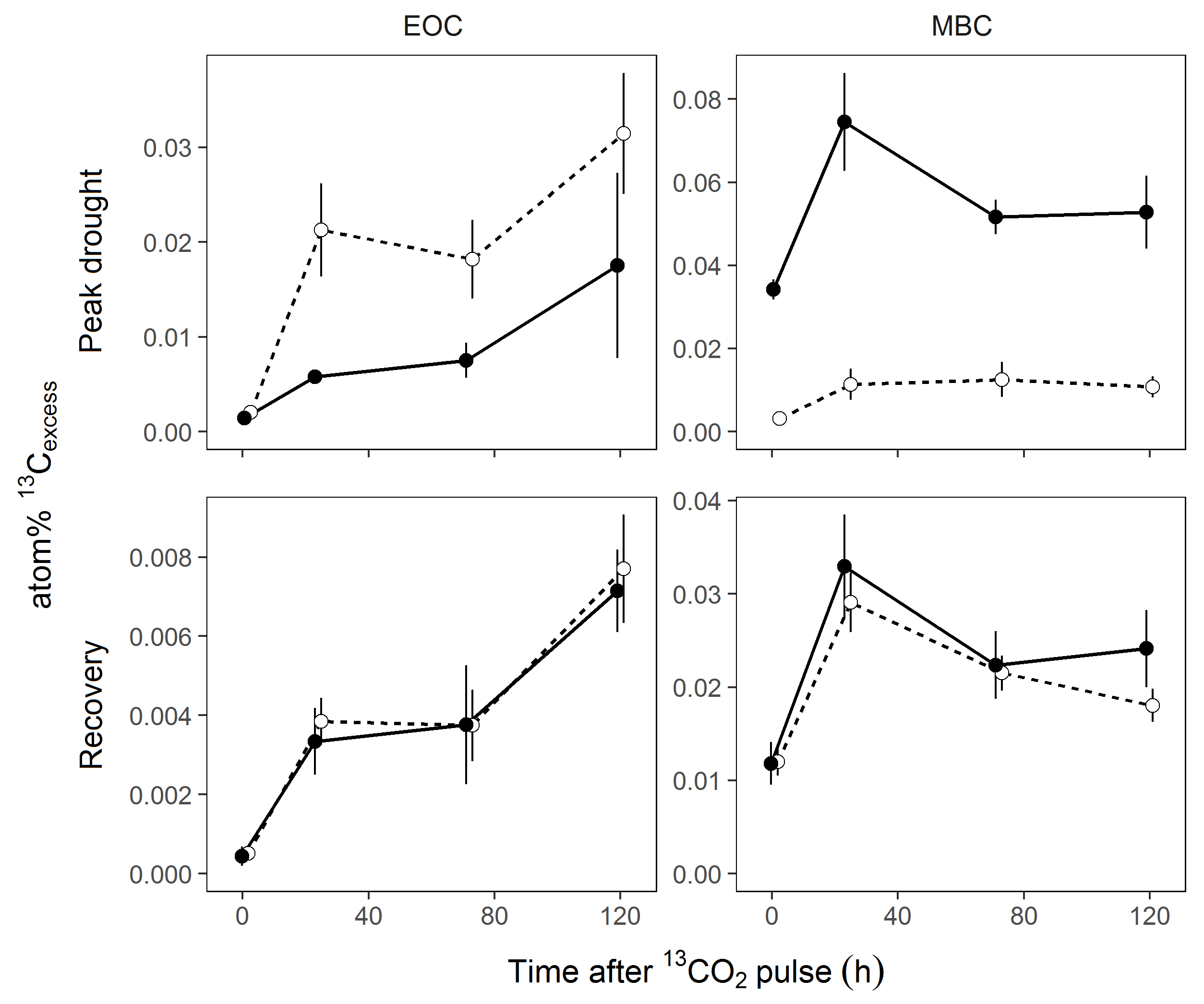


**Supplementary Figure S12.** Dynamics of atom% ^13^C_excess_ in extractable organic carbon (EOC) and microbial biomass carbon (MBC) from soil of control (closed symbols and solid lines) and drought-treated (open symbols and dashed lines) mesocosms at the peak drought and recovery labeling campaigns. Error bars show SE of n = 6 mesocosms.
